# Supplementary material for: A genetically encoded fluorescent biosensor for detecting itaconate with subcellular resolution in living macrophages
Source: Nat Commun. 2022 Nov 4;13:6562. doi: 10.1038/s41467-022-34306-5 (PMC9636186; doi:10.1038/s41467-022-34306-5)
Supplement: Supplementary file 1 — Supplementary information [file 41467_2022_34306_MOESM1_ESM.pdf]

Supplementary Information

This PDF file includes:

Supplementary Fig. 1-7

Supplementary Table 1-4

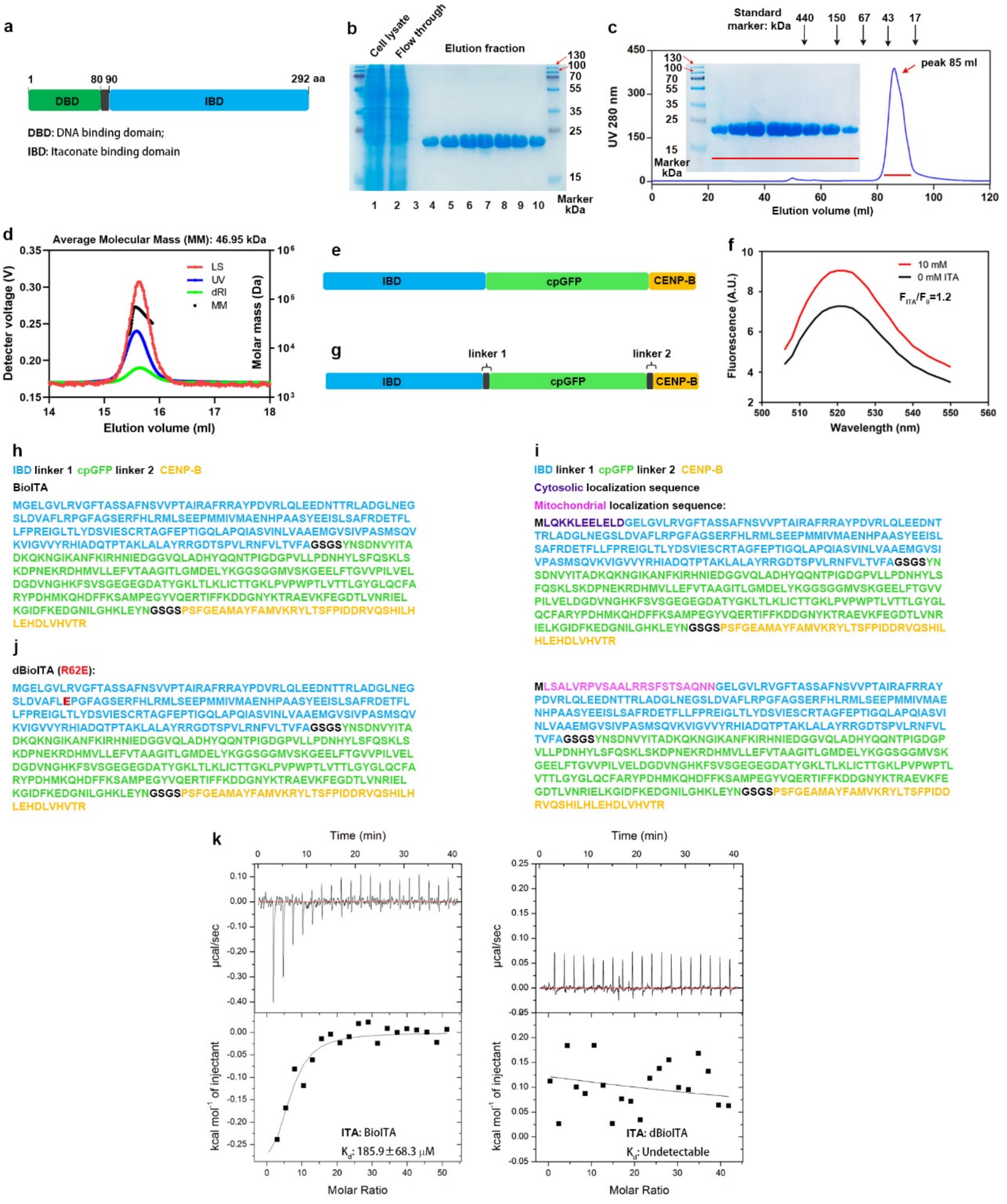

Supplementary Fig. 1: Construction and optimization of itaconate biosensor. a Schematic representation of full-length ItcR with an N-terminal DNA-binding helix-turn-helix motif (residues 1 to 80) and a C-terminal itaconate-binding domain (IBD) (residues 90 to 292). **b** IBD protein tagged with 8 × His was purified by nickel affinity chromatography

from lysate of *E. coli* cells. Elution fraction (lanes 4-10) was separated by 12% sodium dodecyl-sulfate polyacrylamide gel electrophoresis (SDS-PAGE) and stained with *Coomassie* blue. **c** TEV proteinase-digested IBD protein was further purified by size exclusion chromatography on a Superdex 200 column. Elution fraction (red line) was separated by 12% SDS-PAGE and visualized by *Coomassie* blue staining. **d** The molar mass of IBD was measured using an analytical size exclusion column linked to an UV and a multi-angle light scattering (MALS) detector. The UV 280 nm absorption (blue) was displayed together with the molar mass calculated from light scattering intensity (black) versus elution volume. The result calculated from light scattering intensity indicated a molar mass of homodimer (46.95 kDa). **e** Schematic representation of itaconate prototype sensor. IBD, itaconate-binding domain; cpGFP, circularly permuted green fluorescent protein; CENP-B, human centromere protein B. **f** Fluorescence emission scans (excitation at 488 nm) from 250 nM of the itaconate prototype sensor in presence of 10 mM itaconate. F_{ITA} (maxima value from emission peak of the sensor in the presence of 10 mM itaconate) were normalized by F0 (maxima value from emission peak of sensor in the absence of itaconate) and the value of F_{ITA}/ F0 was shown. A.U., arbitrary unit. **g** Schematic representation of itaconate sensors with additional linkers between IBD and cpGFP, cpGFP and CENP-B. **h** Primary amino acid sequence of BioITA. **i** Sequence variants located in N-terminus of

BioITA. **j** Primary amino acid sequence of dBioITA. **k** The binding affinity between the indicated sensors and itaconate was determined by isothermal titration calorimetry (ITC) assay. Binding constant (K_d) of BioITA and dBioITA toward itaconate is shown. The experiments were repeated three times independently with similar results (**b**, **c**, **d**, **f**, **k**). Source data are provided as a Source Data file.

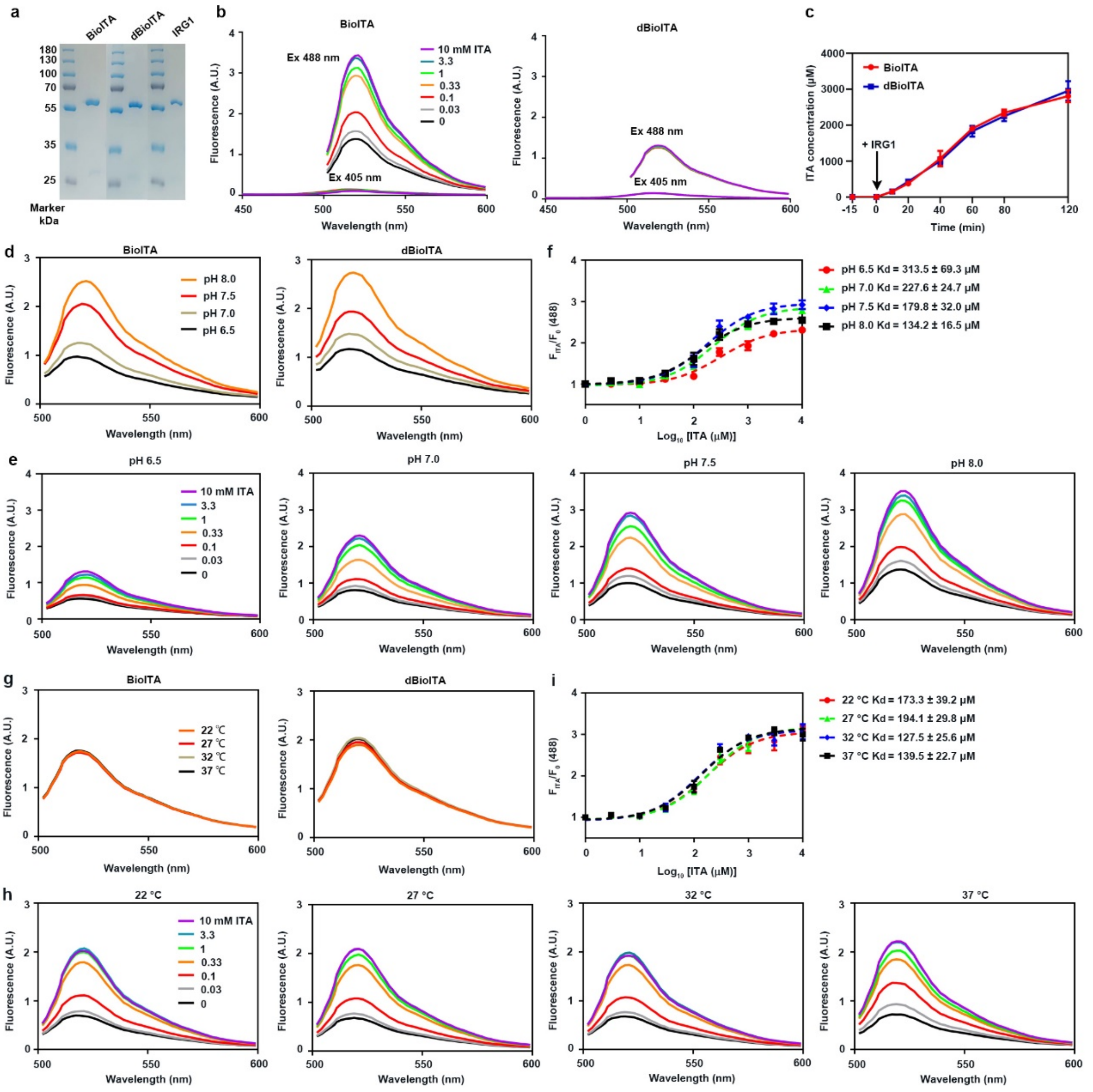

Supplementary Fig. 2: Characterization of BioITA sensor. a Purified BioITA, dBioITA and IRG1 were visualized by *Coomassie*-staining after

separation by SDS-PAGE. dBioITA, dead BioITA. **b** Purified BioITA (left panel) and dBioITA (right panel) were excited either at 405 nm or 488 nm in presence of the indicated concentrations of itaconate, fluorescence emissions were monitored from 450-600 nm or 500-600 nm, respectively. **c** IRG1-catalyzed itaconate production was measured by liquid chromatography–mass spectrometry (LC–MS) at indicated time points post addition of 10 μg active human IRG1 into 100 μl reaction mixture. **d** Fluorescence emission scans (excitation at 488 nm) from BioITA (left panel) and dBioITA (right panel) dissolved in buffer (100 mM HEPES, 150 mM NaCl) with different pH values at 20°C. **e**, **f** Fluorescence emission scans (excitation at 488 nm) from BioITA at indicated pH and itaconate concentrations at 20°C (**e**). Maxima values from emission peaks (excitation at 488 nm) of BioITA versus indicated itaconate concentrations were plotted after normalization to F0 (without itaconate) and the itaconate dissociation constant (K_d) of BioITA at each indicated pH was showed (**f**). **g** Fluorescence emission scans (excitation at 488 nm) from BioITA (left panel) and dBioITA (right panel) dissolved in buffer (100 mM HEPES, 150 mM NaCl, pH [7.4]) at different temperature. **h, i** Fluorescence emission scans (excitation at 488 nm) from BioITA at indicated temperature and itaconate concentrations (**h**). Maxima values from emission peaks (excitation at 488 nm) of BioITA versus indicated itaconate concentrations were plotted after normalization to F0 (without itaconate) and the K_d of

BioITA at each indicated temperature was showed (**i**). The experiments were repeated three times independently with similar results (**a**, **b**, **d**, **e**, **g**, **h**). Data are shown as mean ± SD of three independent experiments (**c**, **f**, **i**). A.U., arbitrary unit (**b**, **d**, **e**, **g**, **h**). Source data are provided as a Source Data file.

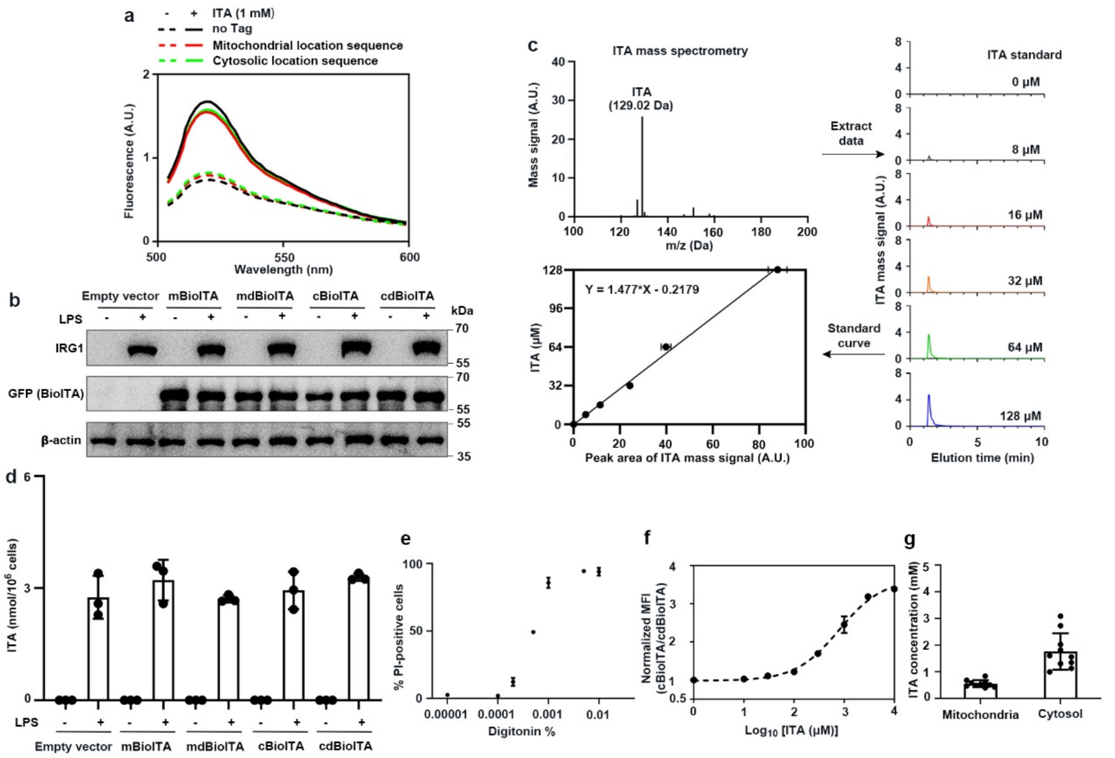

Supplementary Fig. 3: Subcellular itaconate concentrations are detected by BioITA. a Fluorescence emission scans (excitation at 488 nm) from purified BioITAs without (black) or with mitochondrial (red) or cytosolic (green) localization sequences in the presence of 0 (dash line) or 1 mM (solid line) itaconate. **b** RAW264.7 cells without (empty vector) or with expression of indicated BioITAs were stimulated with LPS (10 ng ml^{-1}) for 10 hours. The cell lysates were analyzed by immunoblotting. **c**

Standard curve used for liquid chromatography–mass spectrometry (LC–MS) analysis of itaconate was generated. **d** Intracellular itaconate levels of the cells from (**b**) were determined by LC–MS analyses according to the standard curve from (**c**). **e** RAW264.7 cells were incubated with varying amounts of digitonin for 10 minutes at room temperature. The percentage of cells taking up propidium iodide (PI) was analyzed with flow cytometry (excitation at 561 nm, emission at 670 nm) and shown as a function of digitonin concentration. **f** RAW264.7 cells were permeabilized with 0.001% digitonin and equilibrated with indicated itaconate concentrations for 15 minutes at room temperature. Mean fluorescence intensity (MFI) measured with flow cytometry (excitation at 488 nm, emission at 525 nm) from cBioITA was normalized to the MFI from similarly treated cdBioITA-expressing control RAW264.7 cells. A calibration curve generated by plotting the normalized MFI from cBioITA versus its corresponding equilibrated itaconate concentration was shown. **g** The mBioITA- or cBioITA-expressing RAW264.7 cells were stimulated with LPS for 12 hours. The MFI measured with flow cytometry from mBioITA or cBioITA in non-permeabilized cells was normalized to the MFI from similarly treated mdBioITA- or cdBioITA-expressing control RAW264.7 cells. The concentrations of free itaconate in mitochondria and cytosol of LPS-activated RAW264.7 cells were obtained by interpolating the normalized MFI (10 independent measurements) onto the calibration curve generated

from (**f**). The experiments were repeated three times independently with similar results (**a**, **b**). Data are shown as mean ± SD (n = 3 for **c-f**; n = 10 for **g**). A.U., arbitrary unit (**a**, **c**). Source data are provided as a Source Data file.

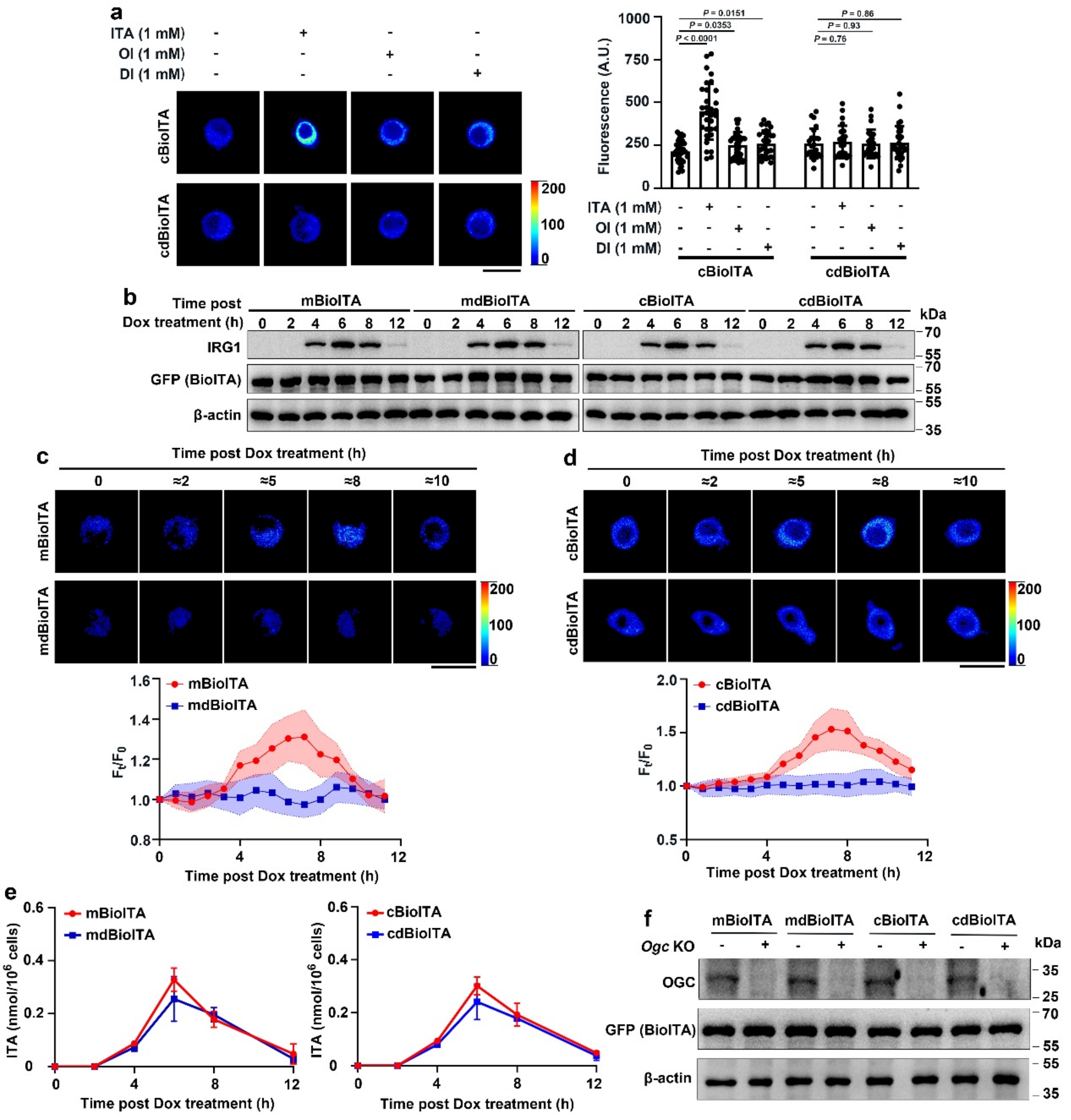

Supplementary Fig. 4: BioITA detects the fluctuation of itaconate in living macrophages. a cBioITA- or cdBioITA-expressing RAW264.7 cells were treated with indicated concentrations of itaconate, 4-octyl itaconate

(OI), or dimethyl-itaconate (DI) for 2 hours. Representative pseudo-color images (left panel) of cBioITA and cdBioITA in RAW264.7 cells were shown. The immunofluorescence intensity in 30 cells from each group was quantitated (right panel). *P* values were determined by the one-way ANOVA. **b-e** Mouse IRG1 was ectopically expressed in RAW264.7 cells stably expressing mitochondria- or cytosol-localized BioITA under the control of a tetracycline-inducible promoter. The cells were treated with doxycycline (Dox) (1 μg ml^{-1}) for 30 min and then the doxycycline was removed by medium change. **b** The cell lysates collected at indicated time points post Dox treatment were analyzed by immunoblotting using indicated antibodies. **c, d** Representative pseudo-color images were captured (**c** and **d**, upper panels) and fluorescence intensity was quantitated at indicated time points post Dox treatment (**c** and **d**, lower panels). Mitochondrial or cytosolic dBioITA served as a control. **e** Intracellular itaconate levels were determined by LC–MS analyses at indicated time points post Dox treatment. **f** Lysates derived from indicated BioITA-expressing RAW264.7 cells without or with *Ogc* knockout (KO) were analyzed by immunoblotting using indicated antibodies. The experiments were repeated three times independently with similar results (**b**, **f**). Data are shown as mean ± SD (n = 30 for **a**; n = 8 for **c**, **d**; n = 3 for **e**). Scale bar, 20 μm (**a**, **c**, **d**). A.U., arbitrary unit (**a**). Dotted lines indicated the mean ± SD (**c** and **d**, lower panels). Source data are provided as a Source

Data file.

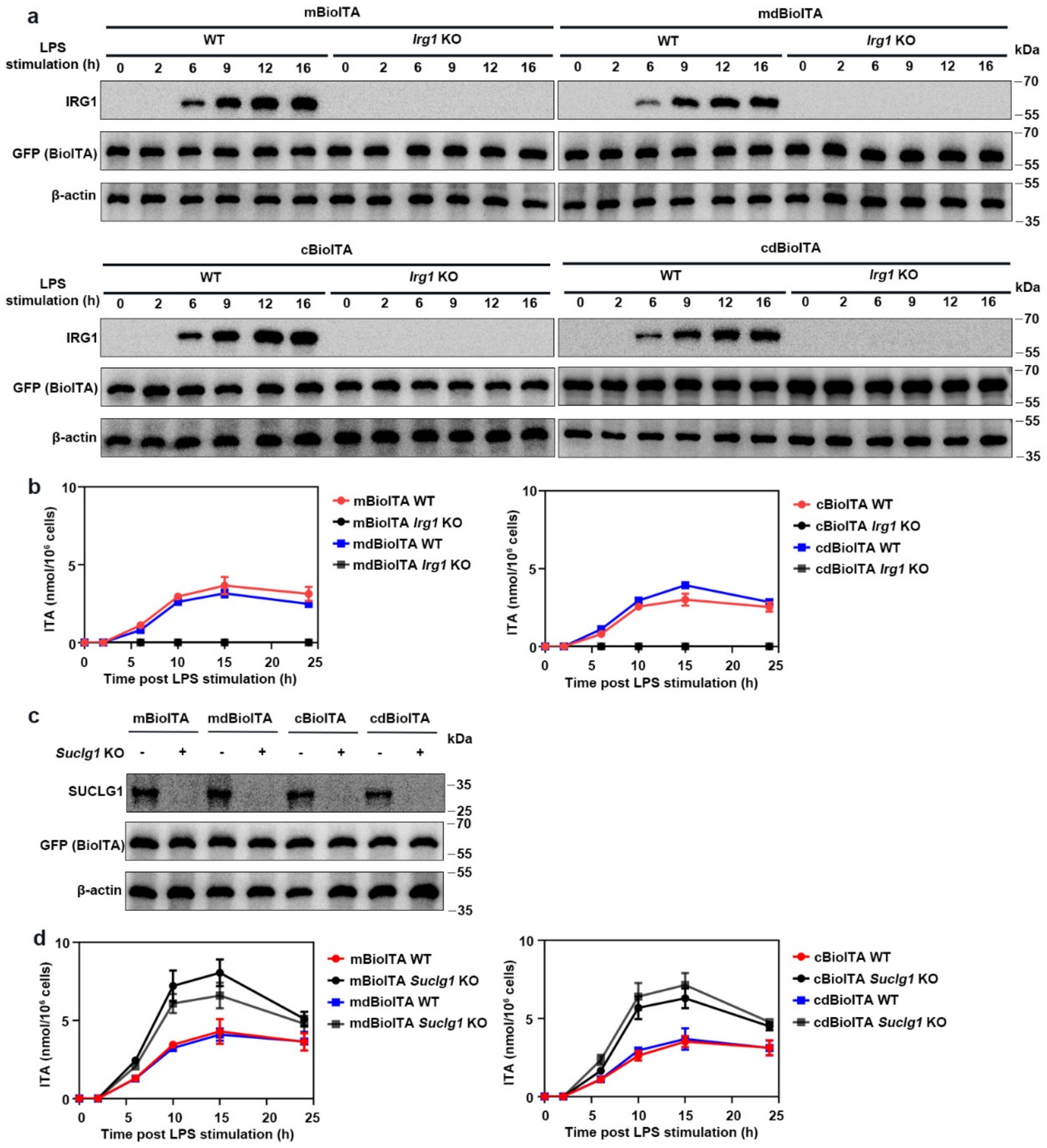

Supplementary Fig. 5: Intracellular itaconate levels are affected by knockout of key genes in itaconate metabolism pathway. a Lysates derived from mitochondrial (upper panel) or cytosolic (lower panel) BioITA-expressing RAW264.7 cells without or with *Irg1* KO were analyzed by immunoblotting using indicated antibodies. **b** Mitochondrial (left panel) or cytosolic (right panel) BioITA-expressing RAW264.7 cells

without or with *Irg1* KO were stimulated with LPS (10 ng ml^{-1}). Intracellular itaconate levels were determined by LC–MS analyses at indicated time points after LPS addition. **c** Lysates derived from indicated BioITA-expressing RAW264.7 cells without or with *Suclg1* KO were analyzed by immunoblotting using indicated antibodies. **d** Mitochondrial (left panel) or cytosolic (right panel) BioITA-expressing RAW264.7 cells without or with *Suclg1* KO were stimulated with LPS (10 ng ml^{-1}). Intracellular itaconate levels were determined by LC–MS analyses at indicated time points after LPS addition. The experiments were repeated three times independently with similar results (**a**, **c**). Data are shown as mean ± SD (n = 3) (**b**, **d**). Source data are provided as a Source Data file.

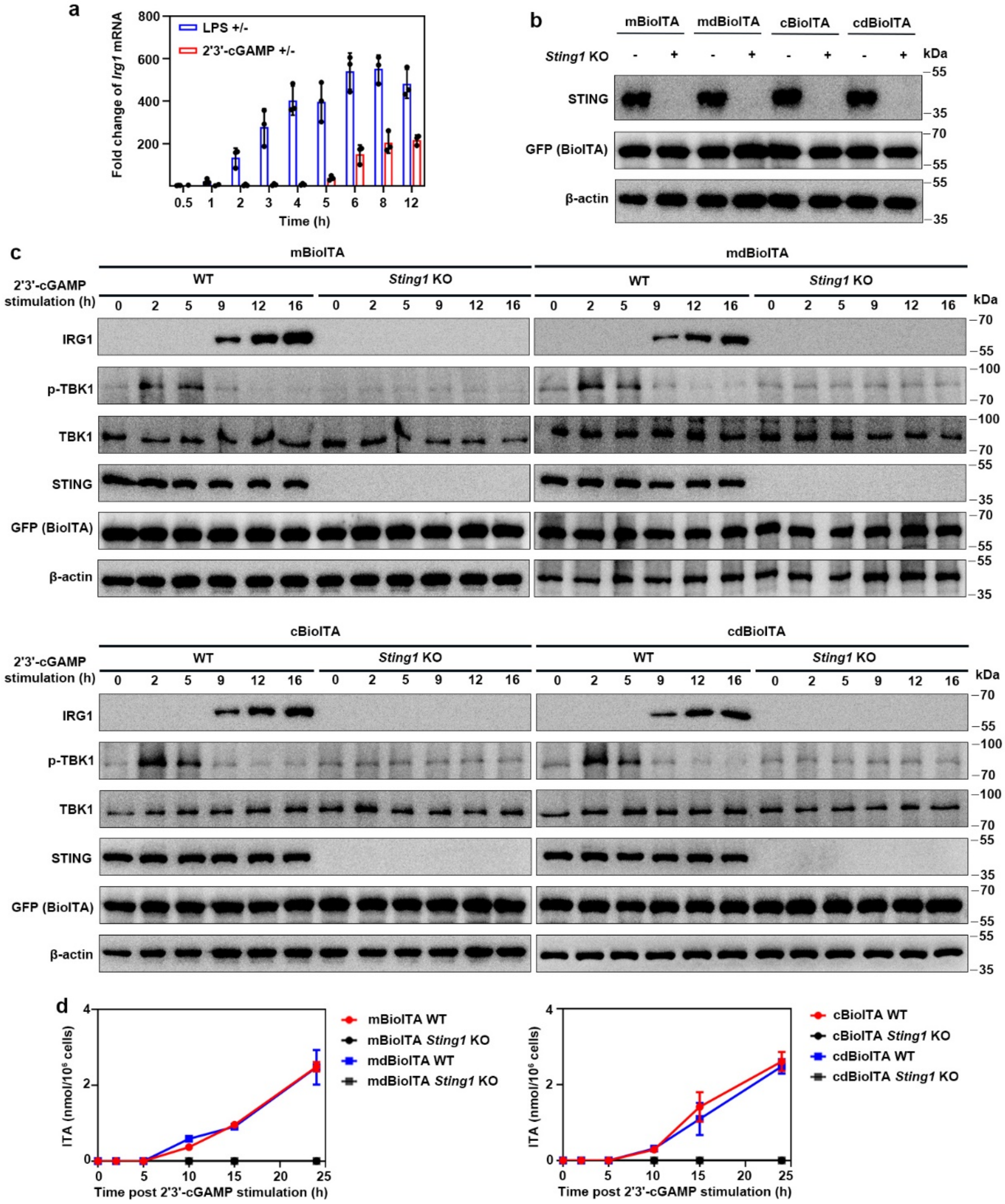

Supplementary Fig. 6: STING activation induces IRG1 expression and itaconate production. a RAW264.7 cells were stimulated with 2'3'-cGAMP (10 μg ml^{-1}) or LPS (10 ng ml^{-1}). Expression levels of *Irg1* mRNA were determined by quantitative PCR (qPCR) at indicated time points post addition of 2'3'-cGAMP or LPS. Data were normalized with β-actin mRNA levels and presented as fold change induced by stimulation of 2'3'-cGAMP

or LPS. Data represent the mean ± SEM of three independent experiments. **b** Lysates derived from indicated BioITA-expressing RAW264.7 cells without or with *Sting1* KO were analyzed by immunoblotting using indicated antibodies. **c, d** Indicated BioITA-expressing RAW264.7 cells without or with *Sting1* KO were treated with 2'3'-cGAMP (10 μg ml^{-1}). Indicated protein levels were detected by immunoblotting (**c**) and intracellular itaconate levels were determined by LC–MS analyses (**d**) at indicated time points after 2'3'-cGAMP addition. Data are shown as mean ± SD (n = 3) (**d**). The experiments were repeated three times independently with similar results (**b**, **c**). Source data are provided as a Source Data file.

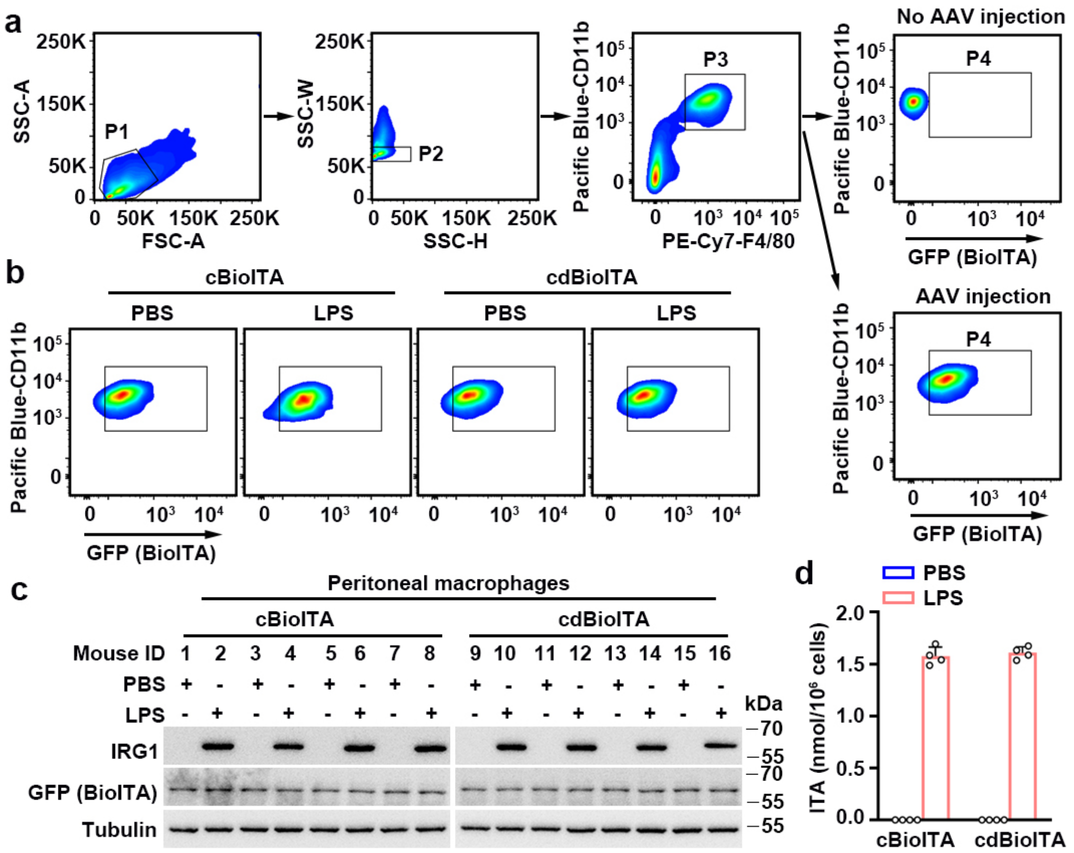

Supplementary Fig. 7: IRG1 expression and itaconate production are

induced in macrophages freshly isolated from LPS-injected mice. a-d Six-week-old female C57BL/6J mice (n = 4 for each group) were intravenously injected with AAV (1×10^{11} viral genomes in 100 µl PBS) coding cBioITA or the control biosensor cdBioITA under the control of a macrophage-specific promoter CD68/150, and then injected with LPS (1 mg kg^{-1}) on day 5 post AAV injection. cBioITA, cytosolic BioITA; cdBioITA, cytosolic dBioITA. **a** Representative gating strategy for flow-cytometry-based analyses of the peritoneal macrophages derived from mice intravenously injected with AAV encoding cBioITA or the control biosensor cdBioITA. Mouse peritoneal cells were selected on the basis of their forward scatter (FSC) and side scatter (SSC) (gate P1). Gate P2 excluded cell doublets present among P1 cells. The P2 cells were subsequently selected for expression of macrophage surface markers $CD11b^+$ $F4/80^+$ (gate P3). Gate P4 selected for expression of GFP (marker for the expression of the cBioITA or cdBioITA). **b** Representative flow cytometry smoothed pseudo-color plots showing fluorescence intensity of cBioITA and cdBioITA in peritoneal macrophages freshly isolated from mice intraperitoneally injected without or with LPS (1 mg kg^{-1}, 8 h). Data are representative of n = 3 biological replicates. **c** IRG1 levels were detected by immunoblotting in peritoneal macrophages freshly isolated from mice (n = 4) intraperitoneally injected without or with LPS (1 mg kg^{-1}, 8 h). **d** Intracellular itaconate levels were determined by LC–MS

analyses in peritoneal macrophages freshly isolated from mice (n = 4) intraperitoneally injected without or with LPS (1 mg kg^{-1}, 8 h). Data are shown as mean ± SEM. Source data are provided as a Source Data file.

Supplementary Table 1. Statistics of X-ray diffraction data and structure refinement

Structure	IBD^{ITA}	SeMet IBD^{ITA}	IBD
PDB code	7W07	7W06	7W08
Diffraction data			
Wavelength (Å)	0.9792	0.9792	0.9792
Space group	C_12_1	C_12_1	$P1$
Cell parameters			
a, b, c (Å)<br>α, β, γ (°)	86.77, 52.58, 49.18<br>90, 123.32, 90	86.77, 52.37, 49.08<br>90, 123.14, 90	68.59, 73.52, 104.03<br>94.24, 105.67, 111.13
Resolution (Å)	50.0-1.48 (1.53-1.48)	50.0-1.36 (1.55-1.50)	50.0-3.25 (3.30-3.25)
Observed reflections	337,573	149,920	129,959
Unique reflections (I/s (I)>0)	27,073	31,747	38,982
Average I/σ(I)	29.0 (2.5)	33.2 (10.1)	7.5 (1.9)
Completeness (%)	94.7 (65.8)	93.9 (65.2)	91.9 (86.9)
R_{merge} (%)	5.0 (70.5)	4.8 (7.4)	16.1 (55.3)
CC ½ (%)	100.0 (85.2)	99.8 (99.3)	96.2 (75.3)
Refinement and structure model			
No. of reflections (Fo>0s(Fo))	27,054	31,734	25,775
Working set	25,684	29,766	23,779
Test set	1,370	1,968	1,996
R_{work}/R_{free} factor (%)	17.9/19.4	17.6/19.5	19.9/23.9
Total atoms	1,744	1,769	12,198
Protein	1,561	1,588	12,198
ITA	9	9	-
Water	169	172	-
Wilson B factor ($Å^2$)	18.4	11.9	51.1
Average B factor ($Å^2$)	23.5	15.0	44.0
Protein	22.8	17.8	44.0
Ligand	24.1	12.9	-
Water	29.6	26.8	-
RMS deviations			
Bond lengths (Å)	0.007	0.008	0.014
Bond angles (°)	0.9	1.3	1.7
Ramachandran plot (%)			
Most favored	98.03	99.5	94.3
Allowed	1.97	0.5	0.6
Disallowed	0	0	0.1

Supplementary Table 2. Sequence information of primers used for molecular cloning and quantitative PCR

Primer number	Purpose	Sequence (5'-3')
1	Dead IBD/BioITA-F	GTCGCTTTCCTGGAGCCAGGCTTTGCTGGC
2	Dead IBD/BioITA-R	GCCAGCAAAGCCTGGCTCCAGGAAAGCGAC
3	Cyto-BioITA N-F	CGGGATCCATGCTCCAGAAAAAGTTAGAAGAATTAGAATTAGATGGAGAACTCGGCGTACTCAGAGTTGGC
4	Mito-BioITA N-F	CGGGATCCATGCTCTCCGCCCTCGTCCGGCCTGTCAGCGCTGCTCTCCGCCGCAGCTTCAGCACCTCGGCCCAGAACAATGGAGAACTCGGCGTACTCAGAG
5	BioITA C-R	CCGCTCGAGTCACCTGGTCACATGAACCAGATCGTGTTCC
6	Cyto-BioITA N-F	GAAGATCTATGCTCCAGAAAAAGTTAGAAGAATTAGAATTAGATGGAGAACTCGGCGTACTCAGAGTTGGC
7	Mito-BioITA N-F	GAAGATCTATGCTCTCCGCCCTCGTCCGGCCTGTCAGCGCTGCTCTCCGCCGCAGCTTCAGCACCTCGGCCCAGAACAATGGAGAACTCGGCGTACTCAGAG
8	BioITA C-R	ACGCGTCGACTCACCTGGTCACATGAACCAGATCGTGTTCC
9	Cyto-BioITA N-F	GGAATTCATGCTCCAGAAAAAGTTAGAAGAATTAGAATTAGATGGAGAACTCGGCGTACTCAGAGTTGGC
10	BioITA C-R	CGGGATCCTCACCTGGTCACATGAACCAGATCGTGTTCC
11	IBD N-F	CGGGATCCATGCAAGCTGCGCAGCGGGCTGTCAGG
12	IBD C-R	CCGCTCGAGTCAAGGAAACACGGTCAGGACAAAGTTTC
13	*IRG1* N-F	CGGGATCCATGATGCTCAAGTCTATCACAGAAAGCTTTGCC
14	*IRG1* C-R	CCGCTCGAGTCAGGAGAGATTTGTGATAGAATTATTACATGCTGGAGAG
15	*Irg1*-tet-on-F	GGAATTCATGATGCTCAAGTCTGTCACAGAGAG
16	*Irg1*-tet-on-R	CGGGATCCTTAGATATTGGTAAACCTGGGCAACG
17	*Irg1*-qPCR-F	CCTGACAGATGGTATCATTCGG
18	*Irg1*-qPCR-R	CTGGAGGTGTTGGAACTGTAG
19	*Actb*-qPCR-F	ACCGTGAAAAGATGACCCAG
20	*Actb*-qPCR-R	GTACGACCAGAGGCATACAG

Supplementary Table 3. Vectors constructed in this study

Vector name	Purpose	Map is provided as a Source Data file
pET-28a IBD	For expression of ITA banding domain of ITCR	Yes
pET-28a dead IBD	For expression of dead ITA banding domain of ITCR	Yes
pET-28a BioITA	For expression of BioITA	Yes
pET-28a dBioITA	For expression of dead BioITA	Yes
pET-28a mBioITA	For expression of mitochondrial BioITA	Yes
pET-28a mdBioITA	For expression of mitochondrial dead BioITA	Yes
pET-28a cBioITA	For expression of cytosolic BioITA	Yes
pET-28a cdBioITA	For expression of cytosolic dead BioITA	Yes
pET-28a hIRG1	For expression of human IRG1	Yes
pLenti cBioITA	For expression of cytosolic BioITA	Yes
pLenti cdBioITA	For expression of cytosolic dead BioITA	Yes
pLenti mBioITA	For expression of mitochondrial BioITA	Yes
pLenti mdBioITA	For expression of mitochondrial dead BioITA	Yes
pLVX-TetOne-Puro-mIRG1	For doxycycline inducible expression of mouse IRG1	Yes
pAAV cBioITA	For expression of cytosolic BioITA	Yes
pAAV cdBioITA	For expression of cytosolic dead BioITA	Yes

Supplementary Table 4. Target sequences of gRNAs

Gene name	Target sequence
Control gRNA	CTTCGAAATGTCCGTTCGGT
Ogc gRNA	TCCGGCCGCGACCTGCCTGG
Irg1 gRNA	CAGCTTGACAAAGTGCCGCG
Suclg1 gRNA	GTATCCCATTCTGTTGCAGG
Sting1 gRNA	ACTTGCGGTTGATCTTACCA
