# Supplementary material for: A genetically encoded fluorescent biosensor for detecting itaconate with subcellular resolution in living macrophages
Source: Nat Commun. 2022 Nov 4;13:6562. doi: 10.1038/s41467-022-34306-5 (PMC9636186; doi:10.1038/s41467-022-34306-5)
Supplement: Supplementary file 3 — Reporting Summary [file 41467_2022_34306_MOESM3_ESM.pdf]

nature portfolio

Corresponding author(s): Xinjian Li

Last updated by author(s): Oct 18, 2022

Reporting Summary

Nature Portfolio wishes to improve the reproducibility of the work that we publish. This form provides structure for consistency and transparency in reporting. For further information on Nature Portfolio policies, see our Editorial Policies and the Editorial Policy Checklist.

Statistics

For all statistical analyses, confirm that the following items are present in the figure legend, table legend, main text, or Methods section.

n/a	Confirmed	
☐	☒	The exact sample size (n) for each experimental group/condition, given as a discrete number and unit of measurement
☐	☒	A statement on whether measurements were taken from distinct samples or whether the same sample was measured repeatedly
☐	☒	The statistical test(s) used AND whether they are one- or two-sided<br>*Only common tests should be described solely by name; describe more complex techniques in the Methods section.*
☐	☒	A description of all covariates tested
☒	☐	A description of any assumptions or corrections, such as tests of normality and adjustment for multiple comparisons
☐	☒	A full description of the statistical parameters including central tendency (e.g. means) or other basic estimates (e.g. regression coefficient) AND variation (e.g. standard deviation) or associated estimates of uncertainty (e.g. confidence intervals)
☐	☒	For null hypothesis testing, the test statistic (e.g. F, t, r) with confidence intervals, effect sizes, degrees of freedom and P value noted<br>*Give P values as exact values whenever suitable.*
☒	☐	For Bayesian analysis, information on the choice of priors and Markov chain Monte Carlo settings
☒	☐	For hierarchical and complex designs, identification of the appropriate level for tests and full reporting of outcomes
☒	☐	Estimates of effect sizes (e.g. Cohen's d, Pearson's r), indicating how they were calculated

Our web collection on statistics for biologists contains articles on many of the points above.

Software and code

Policy information about availability of computer code

Data collection	Microsoft Excel version 2209 Build 16.0.15629.20200 and GraphPad Prism version 8.0.2 was used to draw graphs in the study. Photoshop version CS6 was used to crop images from unprocessed images. Cell images were acquired using an inverted Zeiss LSM980 confocal microscope system. Excitation and emission spectra of the biosensors were acquired using a Synergy H1 spectrofluorometer (BioTek). All the diffraction data sets were collected at BL17U1 and BL18U1 beamlines at the Shanghai Synchrotron Radiation Facility (SSRF). Immunoblots were visualized by a ChemiScope 6000 Exp instrument. Flow cytometry data was collected on a BD LSRFortessa flow cytometer.
Data analysis	X-ray data was processed by the XDS program suite version Jan 31, 2020. Structure was determined by the program AutoSol in PHENIX version 1.18-3855. Structural model building and refinement were carried out using the programs COOT 0.8.9.2 and PHENIX version 1.18-3855. Structure figures were prepared by using the Pymol software version 1.3. IBM SPSS statistics version 23 and GraphPad Prism version 8.0.2 was used to analyze statistical data. FlowJo software version 10.8.1 was used to analyze flow cytometry data. Pixel intensity was quantitated by using Imairs software version 9.0.1. Origin software version 7.0 was used to analyze data from isothermal titration calorimetry (ITC) assay.

For manuscripts utilizing custom algorithms or software that are central to the research but not yet described in published literature, software must be made available to editors and reviewers. We strongly encourage code deposition in a community repository (e.g. GitHub). See the Nature Portfolio guidelines for submitting code & software for further information.

Data

Policy information about availability of data

All manuscripts must include a data availability statement. This statement should provide the following information, where applicable:

- Accession codes, unique identifiers, or web links for publicly available datasets
- A description of any restrictions on data availability
- For clinical datasets or third party data, please ensure that the statement adheres to our policy

The atomic coordinates and structure factors have been deposited in the Protein Data Bank with the accession codes: 7W08 (overall structure of IBD in apo), 7W07 (IBD in complex with itaconate) and 7W06 (SeMet labeled IBD in complex with itaconate). The data supporting the findings of this study are available within the article and its Supplementary Information file. Source data are provided with this paper.

Field-specific reporting

Please select the one below that is the best fit for your research. If you are not sure, read the appropriate sections before making your selection.

☒ Life sciences ☐ Behavioural & social sciences ☐ Ecological, evolutionary & environmental sciences

For a reference copy of the document with all sections, see nature.com/documents/nr-reporting-summary-flat.pdf

Life sciences study design

All studies must disclose on these points even when the disclosure is negative.

Sample size	We used online tools available at http://www.biomath.info/power/ttest.htm to determine the sample or group sizes of the experiments.
Data exclusions	No data were excluded from analysis.
Replication	All the presented experimental results were performed at least three times independently. All attempts at replication were successful.
Randomization	The samples for each experiment were randomized to be examined (No specific methods were used).
Blinding	No blinding was performed due to none of the analyses reported involved procedures that could be influenced by investigator bias.

Reporting for specific materials, systems and methods

We require information from authors about some types of materials, experimental systems and methods used in many studies. Here, indicate whether each material, system or method listed is relevant to your study. If you are not sure if a list item applies to your research, read the appropriate section before selecting a response.

Materials & experimental systems

n/a	Involved in the study	
☐	☒	Antibodies
☐	☒	Eukaryotic cell lines
☒	☐	Palaeontology and archaeology
☐	☒	Animals and other organisms
☒	☐	Human research participants
☒	☐	Clinical data
☒	☐	Dual use research of concern

Methods

n/a	Involved in the study	
☒	☐	ChIP-seq
☐	☒	Flow cytometry
☒	☐	MRI-based neuroimaging

Antibodies

Antibodies used

Rabbit polyclonal antibody recognizing GFP (#G1544, lot: 019M4760V, 1:2000 for immunoblotting) was purchased from Sigma.
Rabbit polyclonal antibody recognizing mouse IRG1 (#17805S, lot: 1, 1:2000 for immunoblotting) was purchased from Cell Signaling Technology.
Mouse monoclonal antibody recognizing β-actin (#T0022-HRP, 1:1000 for immunoblotting) was purchased from Affinity Biosciences.
Rabbit polyclonal antibody recognizing SUCLG1 (#14923-1-AP, lot: 00046682, 1:500 for immunoblotting) was purchased from Proteintech.
Rabbit monoclonal antibody recognizing STING (clone name: D2P2F, #13647, 1:2000 for immunoblotting) was purchased from Cell Signaling Technology.
Rabbit polyclonal antibody recognizing OGC (#K006830P, 1:500 for immunoblotting) was purchased from Beijing Solarbio Science & Technology.

	Rabbit monoclonal antibody recognizing TBK1 (colne name: E8I3G, #38066S, lot: 3, 1:2000 for immunoblotting) was purchased from Cell Signaling Technology.<br>Rabbit monoclonal antibody recognizing p-TBK1 (clone name: D52C2, #5483S, lot: 13, 1:2000 for immunoblotting) was purchased from Cell Signaling Technology.<br>Rabbit monoclonal antibody recognizing α-Tubulin (clone name: P68366, #ab176560, lot: GR177622-63, 1:2000 for immunoblotting) was purchased from Abcam.<br>Rat monoclonal antibody recognizing mouse F4/80 (clone name: BM8, #123113, lot: B173257, 1:500 for immunofluorescence) was purchased from Biolegend.<br>Rat monoclonal antibody recognizing mouse/human CD11b (clone name: M1/70, #101223, lot: B166740, 1:500 for immunofluorescence) was purchased from Biolegend.
Validation	In general, we relied on validation data provided by the manufacturers as well as references in publications.<br>Rabbit polyclonal antibody recognizing GFP (#G1544) for WB, IP. https://www.sigmaaldrich.cn/CN/zh/product/sigma/g1544.<br>Rabbit polyclonal antibody recognizing mouse IRG1 (#17805S) for WB in mouse. https://www.cellsignal.cn/products/primary-antibodies/irg1-antibody/17805?site-search-type=Products&N=4294956287&Ntt=irg1&fromPage=plp.<br>Mouse monoclonal antibody recognizing β-actin (#T0022-HRP) for WB, IHC, IP in human, mouse, Rat, Pig, Zebrafish, Bovine, Sheep, Rabbit, Goat, Guinea pig, Dog, Monkey, Hamster, Chicken, Fish. http://www.affbiotech.com/goods-6281-T0022-beta_Actin_Antibody.html.<br>Rabbit polyclonal antibody recognizing SUCLG1 (#14923-1-AP) for IHC, IP, WB, ELISA in human, mouse, rat. https://www.ptgcn.com/products/SUCLG1-Antibody-14923-1-AP.htm.<br>Rabbit monoclonal antibody recognizing STING (#13647) for WB, IHC, IF/ICC, ELISA in human, mouse. https://www.cellsignal.cn/products/primary-antibodies/sting-d2p2f-rabbit-mab/13647?site-search-type=Products&N=4294956287&Ntt=sting&fromPage=plp.<br>Rabbit polyclonal antibody recognizing OGC (#K006830P) for IHC, WB in human, mouse, rat. https://www.solarbio.com/goods.php?id=62146.<br>Rabbit monoclonal antibody recognizing TBK1 (#38066S) for WB, IP, IF, F in human, mouse, rat. https://www.cellsignal.cn/products/primary-antibodies/tbk1-nak-e8i3g-rabbit-mab/38066?site-search-type=Products&N=4294956287&Ntt=38066&fromPage=plp&_requestid=8600967.<br>Rabbit monoclonal antibody recognizing p-TBK1 (#5483S) for WB, IP, IF, F in human, mouse, rat. https://www.cellsignal.cn/products/primary-antibodies/phospho-tbk1-nak-ser172-d52c2-xp-rabbit-mab/5483?site-search-type=Products&N=4294956287&Ntt=5483&fromPage=plp&_requestid=8601078.<br>Rabbit monoclonal antibody recognizing α-tubulin (#ab176560) for WB, IHC-P, ICC/IF in human, mouse, rat. https://www.abcam.cn/alpha-tubulin-antibody-epr13478b-loading-control-ab176560.html.<br>Rat monoclonal antibody recognizing anti-mouse F4/80 (#123113) for IF. https://www.biolegend.com/en-us/products/pe-cyanine7-anti-mouse-f4-80-antibody-4070.<br>Rat monoclonal antibody recognizing anti-mouse/human CD11b (#101223) for IF. https://www.biolegend.com/en-us/products/pacific-blue-anti-mouse-human-cd11b-antibody-3863.

Eukaryotic cell lines

Policy information about cell lines

Cell line source(s)	RAW 264.7 cells (#TIB-71) were obtained from ATCC.
Authentication	All cell lines used in this study were authenticated with STR profiling.
Mycoplasma contamination	The cell line used in this study were negative for the tests of mycoplasma contamination.
Commonly misidentified lines (See ICLAC register)	No cell lines used in this study were found in the database of commonly misidentified cell lines maintained by ICLAC and NCBI Biosample.

Animals and other organisms

Policy information about studies involving animals; ARRIVE guidelines recommended for reporting animal research

Laboratory animals	Six-week-old female C57BL/6J mice were purchased from GemPharmatech (Nanjing, China). Mice were housed in a pathogen-free environment with the temperature maintained at 23 ± 2°C and relative humidity at 50 to 65% under a 12 h/12 h light/dark cycle with free access to food and water.
Wild animals	No wild animals were used.
Field-collected samples	No field-collected samples were used.
Ethics oversight	The animals were treated in accordance with the Guide for the Care and Use of Laboratory Animals published by the National Academy of Sciences and the National Institutes of Health. The use of animals in this study was approved by the Institutional Animal Care and Use Committee of the Center for Animal Experiments of the Institute of Biophysics, Chinese Academy of Sciences.

Note that full information on the approval of the study protocol must also be provided in the manuscript.

Flow Cytometry

Plots

Confirm that:

☒ The axis labels state the marker and fluorochrome used (e.g. CD4-FITC).

☒ The axis scales are clearly visible. Include numbers along axes only for bottom left plot of group (a 'group' is an analysis of identical markers).

☒ All plots are contour plots with outliers or pseudocolor plots.

☒ A numerical value for number of cells or percentage (with statistics) is provided.

Methodology

Sample preparation	For flow cytometric analysis of the percentage of cells taking up propidium iodide (PI), suspended RAW264.7 cells were incubated with varying amounts of digitonin for 10 minutes at room temperature, the cells were pelleted by centrifugation, resuspended in 1 ml PBS, and passed through a 70-μm filter. The cells were then stained with 1 μg ml−1 of PI just prior to analysis.<br>For flow cytometric measurement of mean fluorescence intensity (MFI) from cBioITA/cdBioITA, suspended RAW264.7 cells without or with digitonin treatment were pelleted by centrifugation, resuspended in 1 ml PBS, and passed through a 70-μm filter, followed by analyzing using a BD LSRFortessa flow cytometer.<br>For flow cytometric analysis of cBioITA/cdBioITA-expressing peritoneal macrophages, mice peritoneal lavage was performed using 10 ml PBS, the cells were pelleted from the lavage fluid by centrifugation, resuspended in 1 ml PBS, and passed through a 70-μm filter. The cells were then stained with 1 μg ml−1 of PE/Cyanine7 anti-mouse F4/80 and Pacific blue anti-mouse/human CD11b antibodies for 20 min at 4 °C. The samples were washed, resuspended in 200 μl PBS, and analyzed using a BD LSRFortessa flow cytometer.
Instrument	BD LSRFortessa flow cytometer
Software	FlowJo software
Cell population abundance	Cell population abundance was determined by the percentage of interested populations in cell pool.
Gating strategy	For flow-cytometry-based analyses of the percentage of cells taking up propidium iodide (PI) or measurement of mean fluorescence intensity (MFI) from cBioITA, RAW264.7 cells were selected on the basis of their forward scatter (FSC) and side scatter (SSC) (gate P1). Gate P2 excluded cell doublets present among P1 cells. The P2 cells were subsequently selected for analyses of the percentage of PI-positive cells or measurement of mean fluorescence intensity (MFI) from cBioITA.<br>For flow-cytometry-based analyses of the peritoneal macrophages derived from mice intravenously injected with AAV encoding cBioITA or the control biosensor cdBioITA, mouse peritoneal cells were selected on the basis of their forward scatter (FSC) and side scatter (SSC) (gate P1). Gate P2 excluded cell doublets present among P1 cells. The P2 cells were subsequently selected for expression of macrophage surface markers CD11b+ F4/80+ (gate P3). Gate P4 selected for expression of GFP (marker for the expression of the cBioITA or cdBioITA).

☒ Tick this box to confirm that a figure exemplifying the gating strategy is provided in the Supplementary Information.
