# Supplementary material for: A genetically encoded fluorescent biosensor for detecting itaconate with subcellular resolution in living macrophages
Source: Nat Commun. 2022 Nov 4;13:6562. doi: 10.1038/s41467-022-34306-5 (PMC9636186; doi:10.1038/s41467-022-34306-5)
Supplement: Supplementary file 4 — Source Data [file 41467_2022_34306_MOESM4_ESM.zip › Structure validation reports/7W08.pdf]

Full wwPDB X-ray Structure Validation Report (i)

Nov 25, 2021 – 07:33 PM JST

PDB ID	:	7W08
Title	:	Itaconate inducible LysR-Type Transcriptional regulator (ITCR) in APO form, Space group P1.
Deposited on	:	2021-11-17
Resolution	:	3.25 Å(reported)

This is a Full wwPDB X-ray Structure Validation Report.

This report is produced by the wwPDB biocuration pipeline after annotation of the structure.

We welcome your comments at *validation@mail.wwpdb.org*
A user guide is available at
https://www.wwpdb.org/validation/2017/XrayValidationReportHelp
with specific help available everywhere you see the (i) symbol.

CONFIDENTIAL VALIDATION REPORT

The following versions of software and data (see references (i)) were used in the production of this report:

MolProbity	:	4.02b-467
Xtriage (Phenix)	:	1.13
EDS	:	2.23.2
Percentile statistics	:	20191225.v01 (using entries in the PDB archive December 25th 2019)
Refmac	:	5.8.0158
CCP4	:	7.0.044 (Gargrove)
Ideal geometry (proteins)	:	Engh & Huber (2001)
Ideal geometry (DNA, RNA)	:	Parkinson et al. (1996)
Validation Pipeline (wwPDB-VP)	:	2.23.2

1 Overall quality at a glance (i)

The following experimental techniques were used to determine the structure:
X-RAY DIFFRACTION

The reported resolution of this entry is 3.25 Å.

Percentile scores (ranging between 0-100) for global validation metrics of the entry are shown in the following graphic. The table shows the number of entries on which the scores are based.

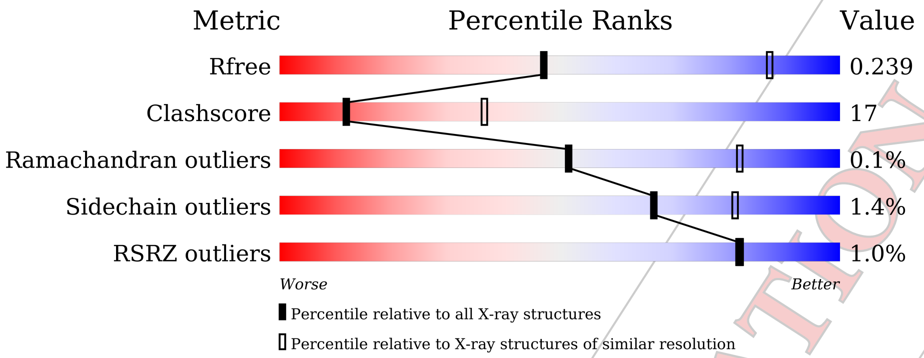

Metric	Whole archive (#Entries)	Similar resolution (#Entries, resolution range(Å))
R_{free}	130704	1191 (3.30-3.22)
Clashscore	141614	1251 (3.30-3.22)
Ramachandran outliers	138981	1229 (3.30-3.22)
Sidechain outliers	138945	1228 (3.30-3.22)
RSRZ outliers	127900	1154 (3.30-3.22)

The table below summarises the geometric issues observed across the polymeric chains and their fit to the electron density. The red, orange, yellow and green segments of the lower bar indicate the fraction of residues that contain outliers for >=3, 2, 1 and 0 types of geometric quality criteria respectively. A grey segment represents the fraction of residues that are not modelled. The numeric value for each fraction is indicated below the corresponding segment, with a dot representing fractions <=5% The upper red bar (where present) indicates the fraction of residues that have poor fit to the electron density. The numeric value is given above the bar.

Mol	Chain	Length	Quality of chain
1	A	292	% 47% 23% 30%
1	B	292	48% 21% • 31%
1	C	292	% 46% 24% • 29%
1	D	292	48% 21% • 30%
1	E	292	% 50% 20% • 29%
1	F	292	% 44% 25% • 30%

Continued on next page...

CONFIDENTIAL VALIDATION REPORT

Continued from previous page...

Mol	Chain	Length	Quality of chain
1	G	292	.% 49% 21% • 29%
1	H	292	.% 49% 20% • 29%

CONFIDENTIAL VALIDATION REPORT

2 Entry composition (i)

There is only 1 type of molecule in this entry. The entry contains 12198 atoms, of which 0 are hydrogens and 0 are deuteriums.

In the tables below, the ZeroOcc column contains the number of atoms modelled with zero occupancy, the AltConf column contains the number of residues with at least one atom in alternate conformation and the Trace column contains the number of residues modelled with at most 2 atoms.

- Molecule 1 is a protein called Transcriptional regulator, LysR family.

Mol	Chain	Residues	Atoms	ZeroOcc	AltConf	Trace
1	C	207	Total C N O S<br>1525 972 262 284 7	0	0	0
1	D	204	Total C N O S<br>1521 971 258 285 7	0	0	0
1	A	204	Total C N O S<br>1469 939 247 276 7	0	0	0
1	B	202	Total C N O S<br>1512 967 257 281 7	0	0	0
1	E	207	Total C N O S<br>1518 971 259 281 7	0	0	0
1	F	205	Total C N O S<br>1561 996 264 294 7	0	1	0
1	G	207	Total C N O S<br>1544 983 264 290 7	0	0	0
1	H	207	Total C N O S<br>1548 989 270 282 7	0	0	0

CONFIDENTIAL VALIDATION REPORT

3 Residue-property plots (i)

These plots are drawn for all protein, RNA, DNA and oligosaccharide chains in the entry. The first graphic for a chain summarises the proportions of the various outlier classes displayed in the second graphic. The second graphic shows the sequence view annotated by issues in geometry and electron density. Residues are color-coded according to the number of geometric quality criteria for which they contain at least one outlier: green = 0, yellow = 1, orange = 2 and red = 3 or more. A red dot above a residue indicates a poor fit to the electron density (RSRZ > 2). Stretches of 2 or more consecutive residues without any outlier are shown as a green connector. Residues present in the sample, but not in the model, are shown in grey.

- Molecule 1: Transcriptional regulator, LysR family

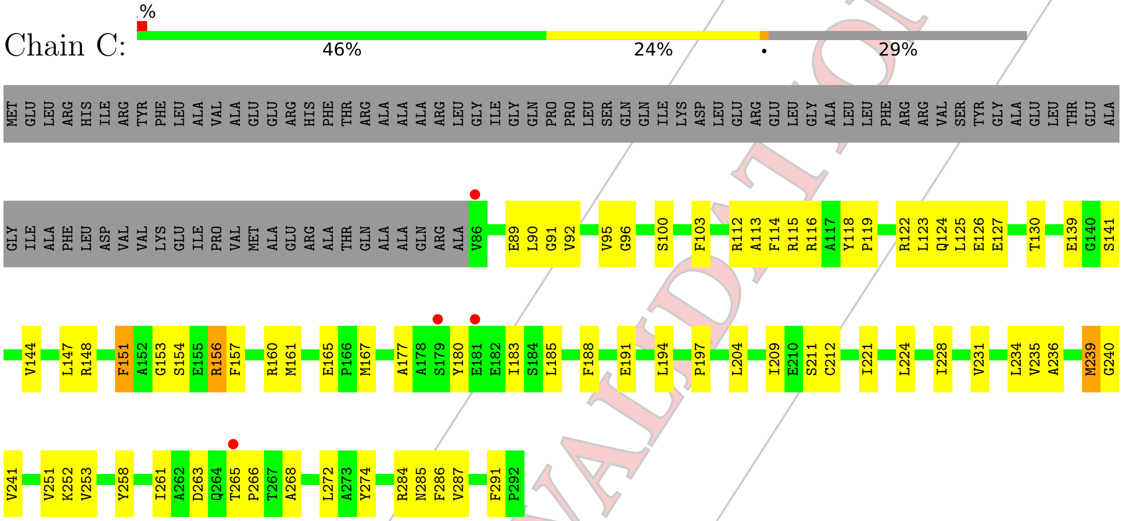

- Molecule 1: Transcriptional regulator, LysR family

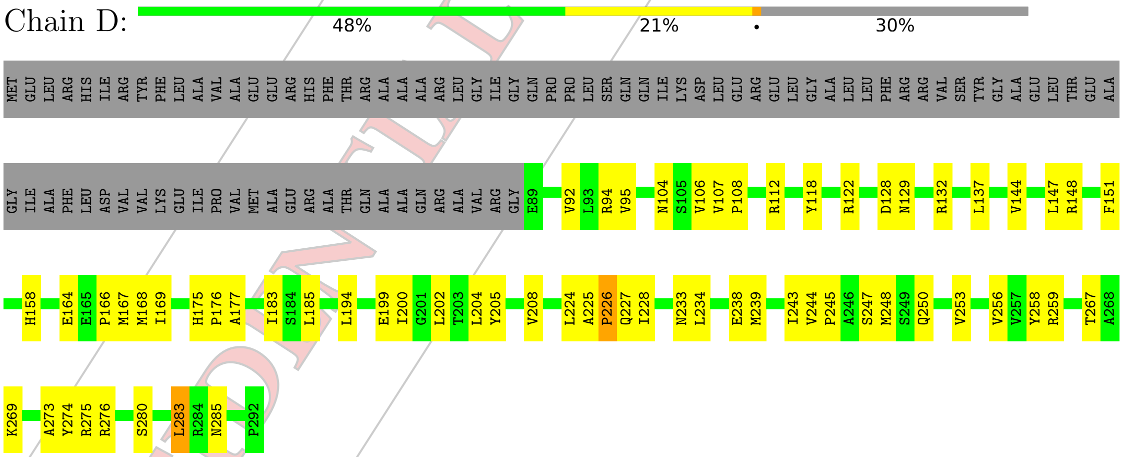

- Molecule 1: Transcriptional regulator, LysR family

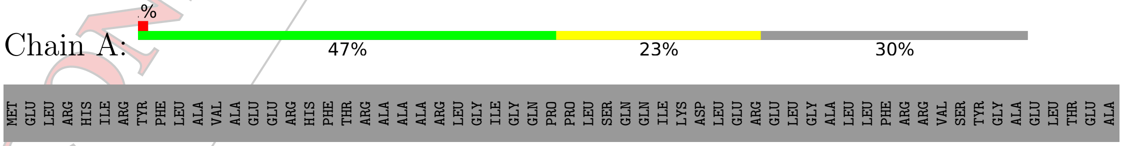

CONFIDENTIAL VALIDATION REPORT

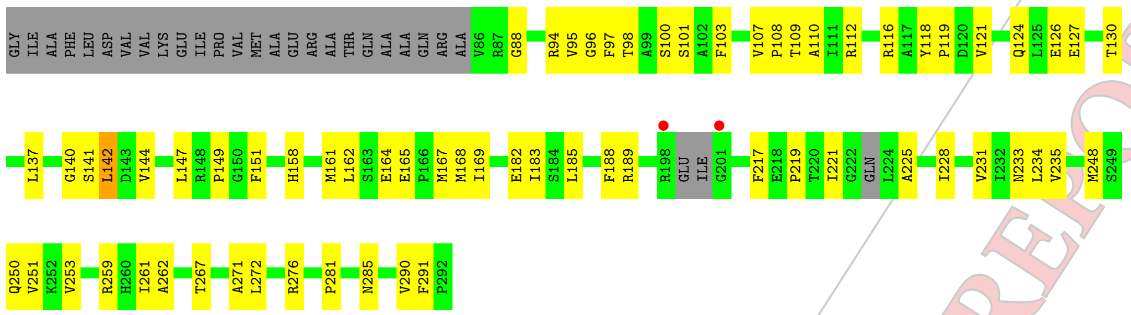

• Molecule 1: Transcriptional regulator, LysR family

Chain B:

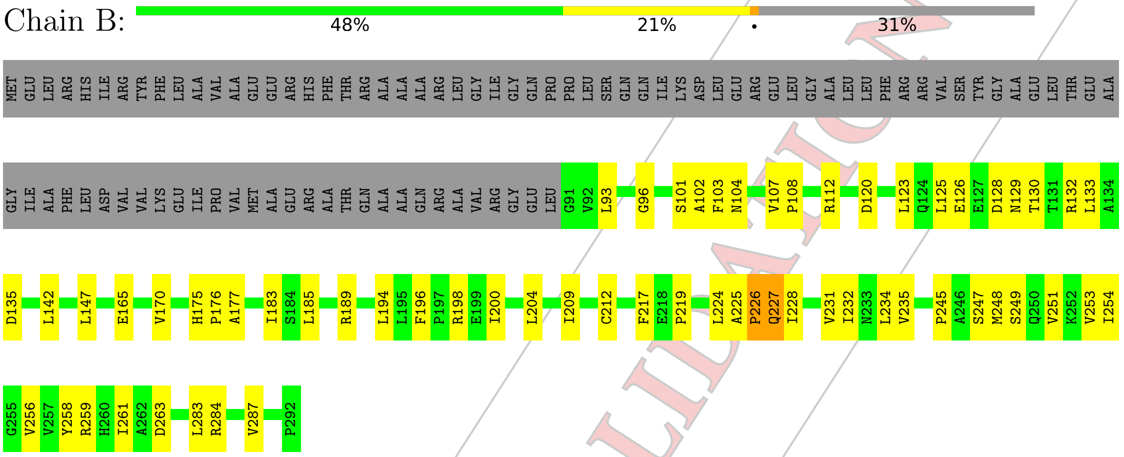

• Molecule 1: Transcriptional regulator, LysR family

Chain E:

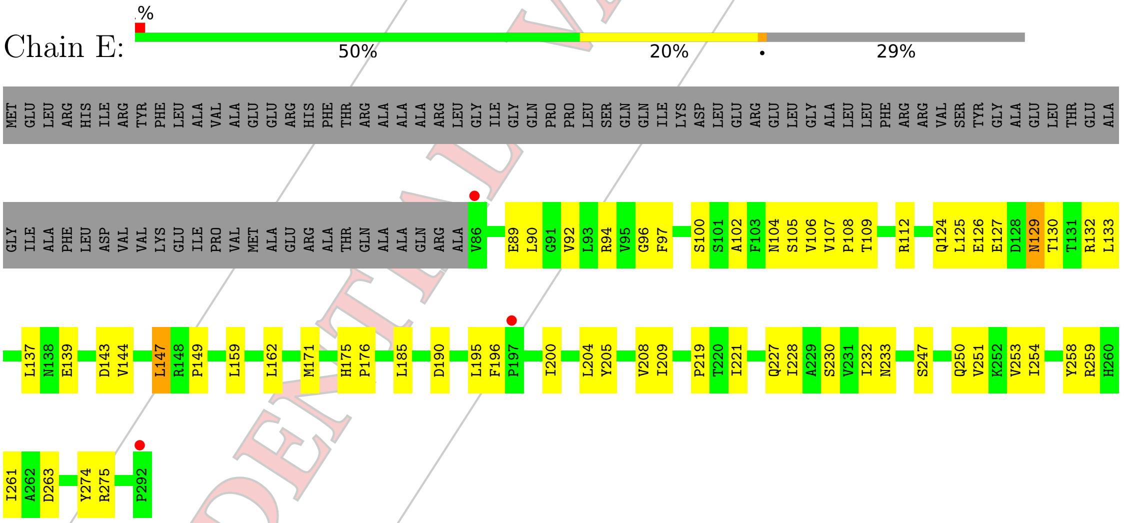

• Molecule 1: Transcriptional regulator, LysR family

Chain F:

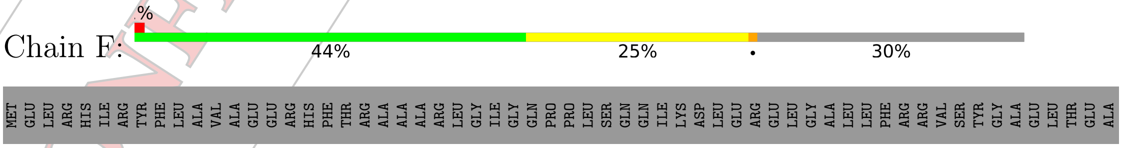

CONFIDENTIAL VALIDATION REPORT

GLY ILE ALA PHE LEU ASP VAL VAL LYS GLU ILE PRO VAL MET ALA GLU ARG ALA THR GLN ALA ALA GLN ARG ALA VAL ARG G88 E89 L90 R94 V95 A102 F103 N104 S105 V106 V107 P108 R115 D120 L123 Q124 L125 T130 T131 R132 D135 G136 L137 S141 L142

D143 V144 L147 E165 M168 I169 Y180 E181 E182 I183 S184 F188 R189 D190 E191 T192 F193 L194 L195 E199 I200 G201 L202 T203 L204 Y205 V208 I209 E210 S211 F217 E218 P219 P226 Q227 I228 V231 I232 N233 L234 V235 A236 A237 E238 M239 V244 P245 M248

S249 Q250 V251 K252 V253 V256 V257 Y258 R259 H260 I261 A262 D263 Q264 T265 P266 T267 A268 K269 L270 A271 L272 R275 L283 R284 F291 P292

• Molecule 1: Transcriptional regulator, LysR family

Chain G: % 49% 21% • 29%

MET GLU LEU ARG HIS ILE ARG TYR PHE LEU ALA VAL ALA GLU GLU ARG HIS PHE THR ARG ALA ALA ALA ARG LEU GLY ILE GLY GLN PRO PRO LEU SER GLN GLN ILE LYS ASP LEU GLU ARG GLU LEU GLY ALA LEU LEU PHE ARG ARG VAL SER TYR GLY ALA GLU LEU THR GLU ALA

GLY ILE ALA PHE LEU ASP VAL VAL LYS GLU ILE PRO VAL MET ALA GLU ARG ALA THR GLN ALA ALA GLN ARG ALA V86 E89 R94 V95 G96 F97 T98 A99 S100 S101 R112 E126 E127 T130 L147 R148 F151 R156 F157 H158 L159 R160 M161 L162 P166

V170 M171 N174 H175 P176 A177 A178 I183 F188 E191 F196 P197 R198 G201 Y205 V208 I209 C212 R213 F217 E218 P219 L224 A225 I228 V231 V235 A236 I243 V244 P245 A246 S249 Q250 V251 K252 V253 V256 D263 Q264 T265 P266

T267 A268 K269 A273 Y274 R275 D278 N285 T289 P292

• Molecule 1: Transcriptional regulator, LysR family

Chain H: % 49% 20% • 29%

MET GLU LEU ARG HIS ILE ARG TYR PHE LEU ALA VAL ALA GLU GLU ARG HIS PHE THR ARG ALA ALA ALA ARG LEU GLY ILE GLY GLN PRO PRO LEU SER GLN GLN ILE LYS ASP LEU GLU ARG GLU LEU GLY ALA LEU LEU PHE ARG ARG VAL SER TYR GLY ALA GLU LEU THR GLU ALA

GLY ILE ALA PHE LEU ASP VAL VAL LYS GLU ILE PRO VAL MET ALA GLU ARG ALA THR GLN ALA ALA GLN ARG ALA V86 R94 V95 G96 S100 A110 I111 L123 E126 N129 T130 T131 L142 D143 V144 L147 S154 H158 E164 E165 P166 M167 M168 I169

S179 Y180 E181 E182 I183 R189 L195 R198 E199 I200 G201 L202 T203 L204 Y205 C212 R213 F217 E218 P219 Q223 L224 A225 P226 Q227 I228 A229 S230 V231 I232 N233 L234 V235 E238 P245 A246 S247 M248 S249 Q250 V251 K252 V253 R259 Q264 T267 A268 K269

L272 A273 R276 V282 L283 L288 T289 V290 F291 P292

CONFIDENTIAL VALIDATION REPORT

4 Data and refinement statistics (i)

Property	Value	Source
Space group	P 1	Depositor
Cell constants<br>a, b, c, α, β, γ	68.59Å 73.52Å 104.03Å<br>94.24° 105.67° 111.13°	Depositor
Resolution (Å)	32.96 – 3.25<br>40.37 – 3.25	Depositor<br>EDS
% Data completeness<br>(in resolution range)	91.4 (32.96-3.25)<br>87.2 (40.37-3.25)	Depositor<br>EDS
R_{merge}	0.17	Depositor
R_{sym}	(Not available)	Depositor
$< I/\sigma(I) >$ [1]	1.84 (at 3.25Å)	Xtriage
Refinement program	PHENIX 1.18_3855	Depositor
R, R_{free}	0.199 , 0.239<br>0.199 , 0.239	Depositor<br>DCC
R_{free} test set	2000 reflections (7.75%)	wwPDB-VP
Wilson B-factor (Å^2)	51.1	Xtriage
Anisotropy	0.298	Xtriage
Bulk solvent k_{sol}(e/Å^3), B_{sol}(Å^2)	0.35 , 49.6	EDS
L-test for twinning[2]	$< \|L\| > = 0.48$, $< L^2 > = 0.31$	Xtriage
Estimated twinning fraction	No twinning to report.	Xtriage
F_o,F_c correlation	0.90	EDS
Total number of atoms	12198	wwPDB-VP
Average B, all atoms (Å^2)	44.0	wwPDB-VP

Xtriage's analysis on translational NCS is as follows: *The largest off-origin peak in the Patterson function is 5.49% of the height of the origin peak. No significant pseudotranslation is detected.*

[1]Intensities estimated from amplitudes.

[2]Theoretical values of $< |L| >$, $< L^2 >$ for acentric reflections are 0.5, 0.333 respectively for untwinned datasets, and 0.375, 0.2 for perfectly twinned datasets.

CONFIDENTIAL VALIDATION REPORT

5 Model quality (i)

5.1 Standard geometry (i)

The Z score for a bond length (or angle) is the number of standard deviations the observed value is removed from the expected value. A bond length (or angle) with $|Z| > 5$ is considered an outlier worth inspection. RMSZ is the root-mean-square of all Z scores of the bond lengths (or angles).

Mol	Chain	Bond lengths		Bond angles	
		RMSZ	$\#\|Z\| > 5$	RMSZ	$\#\|Z\| > 5$
1	A	0.59	0/1495	0.87	3/2038 (0.1%)
1	B	0.63	0/1542	0.96	2/2101 (0.1%)
1	C	0.65	1/1554 (0.1%)	0.89	1/2119 (0.0%)
1	D	0.67	0/1551	0.97	3/2114 (0.1%)
1	E	0.57	0/1549	0.86	1/2114 (0.0%)
1	F	0.71	0/1592	0.96	5/2167 (0.2%)
1	G	0.72	2/1575 (0.1%)	0.90	4/2145 (0.2%)
1	H	0.72	0/1578	0.99	8/2149 (0.4%)
All	All	0.66	3/12436 (0.0%)	0.93	27/16947 (0.2%)

All (3) bond length outliers are listed below:

Mol	Chain	Res	Type	Atoms	Z	Observed(Å)	Ideal(Å)
1	G	245	PRO	N-CA	12.09	1.67	1.47
1	C	212	CYS	CB-SG	-5.47	1.72	1.81
1	G	244	VAL	C-N	5.20	1.44	1.34

All (27) bond angle outliers are listed below:

Mol	Chain	Res	Type	Atoms	Z	Observed(^{o})	Ideal(^{o})
1	B	226	PRO	N-CA-CB	12.59	118.41	103.30
1	D	283	LEU	CB-CG-CD1	-10.75	92.72	111.00
1	H	225	ALA	CB-CA-C	-7.90	98.25	110.10
1	D	168	MET	CB-CG-SD	-7.60	89.60	112.40
1	H	223	GLN	CB-CA-C	-7.41	95.58	110.40
1	H	224	LEU	N-CA-C	-7.10	91.83	111.00
1	C	239	MET	CB-CG-SD	-7.06	91.23	112.40
1	H	225	ALA	N-CA-CB	6.70	119.47	110.10
1	G	162	LEU	CB-CG-CD2	-6.51	99.94	111.00
1	H	225	ALA	N-CA-C	-6.48	93.50	111.00
1	A	168	MET	CG-SD-CE	6.43	110.49	100.20
1	H	202	LEU	CB-CG-CD2	-6.24	100.39	111.00
1	G	245	PRO	CA-N-CD	-6.11	102.94	111.50

Continued on next page...

CONFIDENTIAL VALIDATION REPORT

Continued from previous page...

Mol	Chain	Res	Type	Atoms	Z	Observed(°)	Ideal(°)
1	A	142	LEU	CA-CB-CG	6.10	129.33	115.30
1	F	183	ILE	CG1-CB-CG2	-6.05	98.09	111.40
1	G	245	PRO	N-CA-C	-5.95	96.63	112.10
1	E	147	LEU	CB-CG-CD2	-5.93	100.92	111.00
1	H	198	ARG	NE-CZ-NH2	5.68	123.14	120.30
1	F	272	LEU	CB-CG-CD1	-5.66	101.39	111.00
1	D	226	PRO	N-CA-CB	5.63	110.06	103.30
1	B	227	GLN	CB-CA-C	-5.50	99.40	110.40
1	H	224	LEU	CB-CA-C	-5.50	99.75	110.20
1	F	234	LEU	CB-CG-CD1	-5.47	101.70	111.00
1	A	167	MET	CG-SD-CE	5.38	108.81	100.20
1	F	90	LEU	CB-CG-CD2	-5.28	102.02	111.00
1	G	94	ARG	NE-CZ-NH2	-5.22	117.69	120.30
1	F	252	LYS	CD-CE-NZ	5.10	123.43	111.70

There are no chirality outliers.

There are no planarity outliers.

5.2 Too-close contacts (i)

In the following table, the Non-H and H(model) columns list the number of non-hydrogen atoms and hydrogen atoms in the chain respectively. The H(added) column lists the number of hydrogen atoms added and optimized by MolProbity. The Clashes column lists the number of clashes within the asymmetric unit, whereas Symm-Clashes lists symmetry-related clashes.

Mol	Chain	Non-H	H(model)	H(added)	Clashes	Symm-Clashes
1	A	1469	0	1391	50	0
1	B	1512	0	1495	63	0
1	C	1525	0	1493	69	0
1	D	1521	0	1491	56	0
1	E	1518	0	1468	49	0
1	F	1561	0	1550	69	0
1	G	1544	0	1503	48	0
1	H	1548	0	1546	68	0
All	All	12198	0	11937	417	0

The all-atom clashscore is defined as the number of clashes found per 1000 atoms (including hydrogen atoms). The all-atom clashscore for this structure is 17.

All (417) close contacts within the same asymmetric unit are listed below, sorted by their clash magnitude.

CONFIDENTIAL VALIDATION REPORT

Atom-1	Atom-2	Interatomic distance (Å)	Clash overlap (Å)
1:G:245:PRO:N	1:G:245:PRO:CA	1.67	1.47
1:D:225:ALA:HB1	1:B:225:ALA:HB1	1.24	1.16
1:H:225:ALA:CB	1:H:226:PRO:HD2	1.70	1.16
1:H:181:GLU:HA	1:H:259:ARG:HH22	1.17	1.05
1:H:225:ALA:HB1	1:H:226:PRO:HD2	1.38	1.04
1:F:199:GLU:OE2	1:F:231:VAL:HG12	1.59	1.00
1:C:139:GLU:OE1	1:B:198:ARG:HG3	1.61	0.99
1:H:225:ALA:CB	1:H:226:PRO:CD	2.40	0.99
1:C:148:ARG:HD2	1:C:204:LEU:CD1	1.94	0.97
1:H:225:ALA:HB3	1:H:226:PRO:HD2	1.43	0.97
1:D:202:LEU:HD21	1:A:140:GLY:HA3	1.46	0.96
1:C:124:GLN:HE22	1:B:225:ALA:HB2	1.29	0.96
1:H:225:ALA:HB1	1:H:226:PRO:CD	1.95	0.95
1:F:130:THR:HG23	1:F:147:LEU:HB2	1.49	0.94
1:D:225:ALA:HB1	1:B:225:ALA:CB	1.97	0.93
1:C:124:GLN:NE2	1:B:225:ALA:HB2	1.85	0.90
1:H:130:THR:HG23	1:H:147:LEU:HB2	1.55	0.88
1:E:149:PRO:HB3	1:E:159:LEU:HD21	1.54	0.88
1:C:92:VAL:HG21	1:B:224:LEU:CD1	2.04	0.87
1:G:253:VAL:HA	1:H:250:GLN:HG2	1.57	0.87
1:E:130:THR:HG23	1:E:147:LEU:HB2	1.57	0.86
1:D:227:GLN:HE21	1:A:141:SER:HB2	1.41	0.86
1:F:228:ILE:HD11	1:F:234:LEU:HD11	1.56	0.85
1:A:164:GLU:OE1	1:A:267:THR:OG1	1.93	0.83
1:A:130:THR:HG23	1:A:147:LEU:HB2	1.60	0.83
1:H:181:GLU:HA	1:H:259:ARG:NH2	1.94	0.82
1:D:225:ALA:CB	1:B:225:ALA:HB1	2.09	0.80
1:E:124:GLN:NE2	1:F:226:PRO:HG3	1.98	0.79
1:B:200:ILE:HG23	1:B:204:LEU:HD23	1.64	0.79
1:E:129:ASN:O	1:E:133:LEU:HG	1.83	0.78
1:B:132:ARG:HA	1:B:135:ASP:HB2	1.63	0.78
1:D:225:ALA:CB	1:B:225:ALA:CB	2.62	0.77
1:E:92:VAL:HG21	1:H:224:LEU:HD13	1.65	0.77
1:C:92:VAL:HG21	1:B:224:LEU:HD11	1.66	0.76
1:C:139:GLU:HG2	1:C:141:SER:HB3	1.65	0.76
1:D:148:ARG:HD2	1:D:204:LEU:HD13	1.69	0.75
1:B:196:PHE:CD2	1:B:231:VAL:HG22	2.22	0.75
1:A:272:LEU:HD23	1:A:291:PHE:HE1	1.51	0.75
1:E:124:GLN:HE22	1:F:226:PRO:HG3	1.50	0.75
1:G:245:PRO:N	1:G:245:PRO:C	2.40	0.74
1:D:227:GLN:NE2	1:A:141:SER:HB2	2.02	0.74
1:G:98:THR:HG1	1:G:101:SER:HG	0.74	0.73

Continued on next page...

CONFIDENTIAL VALIDATION REPORT

Continued from previous page...

Atom-1	Atom-2	Interatomic distance (Å)	Clash overlap (Å)
1:F:183:ILE:HG22	1:F:184:SER:H	1.52	0.73
1:G:196:PHE:HE2	1:G:205:TYR:HB2	1.54	0.72
1:E:261:ILE:HG22	1:E:263:ASP:H	1.55	0.72
1:F:209:ILE:HD13	1:F:219:PRO:HG3	1.72	0.72
1:B:198:ARG:CZ	1:B:227:GLN:HB3	2.19	0.72
1:B:120:ASP:OD2	1:H:282:VAL:HG23	1.91	0.71
1:G:100:SER:HB3	1:G:228:ILE:HD12	1.72	0.70
1:G:196:PHE:CE2	1:G:205:TYR:HB2	2.26	0.70
1:E:137:LEU:O	1:E:275:ARG:NH1	2.25	0.70
1:H:129:ASN:ND2	1:H:131:THR:OG1	2.24	0.70
1:A:169:ILE:HB	1:A:261:ILE:HD11	1.74	0.69
1:C:100:SER:HB3	1:C:228:ILE:HG13	1.75	0.69
1:D:94:ARG:NH1	1:E:190:ASP:OD1	2.26	0.69
1:G:171:MET:HE1	1:G:177:ALA:HB3	1.75	0.68
1:F:199:GLU:OE2	1:F:231:VAL:CG1	2.37	0.68
1:G:212:CYS:SG	1:G:219:PRO:HG3	2.34	0.67
1:E:149:PRO:HB3	1:E:159:LEU:CD2	2.24	0.66
1:C:272:LEU:HD23	1:C:291:PHE:HE2	1.61	0.66
1:C:148:ARG:CD	1:C:204:LEU:HD11	2.26	0.66
1:C:148:ARG:HD2	1:C:204:LEU:HD11	1.77	0.65
1:C:153:GLY:O	1:C:156:ARG:HD3	1.97	0.65
1:C:265:THR:N	1:C:266:PRO:HD3	2.11	0.65
1:D:147:LEU:HD13	1:D:151:PHE:HE2	1.61	0.64
1:E:112:ARG:HD3	1:F:238:GLU:HG3	1.79	0.64
1:B:212:CYS:HB3	1:B:219:PRO:HD3	1.80	0.64
1:C:92:VAL:HG21	1:B:224:LEU:HD13	1.79	0.64
1:C:148:ARG:CD	1:C:204:LEU:CD1	2.74	0.63
1:C:185:LEU:HG	1:C:261:ILE:HG21	1.80	0.63
1:G:265:THR:O	1:G:265:THR:OG1	2.08	0.63
1:H:225:ALA:O	1:H:226:PRO:C	2.36	0.63
1:B:130:THR:HG23	1:B:147:LEU:HB2	1.79	0.63
1:C:124:GLN:HE22	1:B:225:ALA:CB	2.06	0.63
1:B:103:PHE:O	1:B:247:SER:HB3	1.99	0.63
1:E:139:GLU:OE2	1:H:198:ARG:NH2	2.23	0.63
1:B:198:ARG:NH1	1:B:227:GLN:HB3	2.14	0.63
1:F:204:LEU:HD21	1:F:245:PRO:HG3	1.80	0.62
1:F:245:PRO:HD2	1:F:248:MET:HG3	1.81	0.62
1:E:102:ALA:O	1:F:233:ASN:ND2	2.32	0.62
1:F:200:ILE:HG22	1:F:204:LEU:HB3	1.82	0.62
1:E:196:PHE:HZ	1:E:204:LEU:HB3	1.64	0.61
1:F:189:ARG:HA	1:F:217:PHE:CE2	2.36	0.61

Continued on next page...

CONFIDENTIAL VALIDATION REPORT

Continued from previous page...

Atom-1	Atom-2	Interatomic distance (Å)	Clash overlap (Å)
1:F:132:ARG:HG2	1:F:132:ARG:HH21	1.65	0.60
1:F:94:ARG:HG3	1:F:124:GLN:HB3	1.82	0.60
1:H:164:GLU:HG2	1:H:267:THR:OG1	2.01	0.60
1:H:199:GLU:HG3	1:H:229:ALA:HB1	1.84	0.60
1:C:148:ARG:HD2	1:C:204:LEU:HD13	1.80	0.60
1:G:160:ARG:HH11	1:G:160:ARG:HG3	1.67	0.60
1:H:200:ILE:HG22	1:H:204:LEU:HB3	1.83	0.60
1:C:92:VAL:CG2	1:B:224:LEU:CD1	2.79	0.59
1:H:94:ARG:NH2	1:H:143:ASP:OD1	2.35	0.59
1:H:96:GLY:HA2	1:H:126:GLU:O	2.01	0.59
1:D:258:TYR:O	1:D:259:ARG:HD2	2.03	0.59
1:C:144:VAL:CG2	1:C:272:LEU:HD11	2.33	0.59
1:H:158:HIS:CE1	1:H:276:ARG:HA	2.38	0.59
1:E:149:PRO:CB	1:E:159:LEU:HD21	2.29	0.59
1:D:224:LEU:CB	1:A:124:GLN:HG3	2.33	0.59
1:B:224:LEU:HD23	1:B:224:LEU:H	1.68	0.58
1:H:100:SER:HB3	1:H:199:GLU:O	2.03	0.58
1:A:96:GLY:HA2	1:A:126:GLU:O	2.02	0.58
1:H:181:GLU:CA	1:H:259:ARG:HH22	2.05	0.58
1:D:158:HIS:CE1	1:D:276:ARG:HH21	2.21	0.58
1:H:251:VAL:HG12	1:H:253:VAL:HG23	1.85	0.58
1:A:219:PRO:O	1:A:221:ILE:HG13	2.04	0.58
1:C:95:VAL:HB	1:C:125:LEU:HD23	1.85	0.58
1:E:129:ASN:ND2	1:E:200:ILE:O	2.37	0.58
1:D:118:TYR:OH	1:D:285:ASN:ND2	2.37	0.57
1:E:139:GLU:CD	1:H:198:ARG:HH21	2.05	0.57
1:D:247:SER:O	1:D:250:GLN:NE2	2.30	0.57
1:D:107:VAL:HB	1:D:108:PRO:HD3	1.86	0.57
1:A:109:THR:HG23	1:A:112:ARG:HH21	1.70	0.57
1:D:202:LEU:HD21	1:A:140:GLY:CA	2.27	0.57
1:B:133:LEU:HD22	1:B:142:LEU:HD11	1.86	0.57
1:H:276:ARG:HG2	1:H:276:ARG:HH11	1.69	0.57
1:A:116:ARG:O	1:A:119:PRO:HD3	2.05	0.56
1:D:175:HIS:ND1	1:D:176:PRO:HD2	2.21	0.56
1:G:171:MET:HE3	1:G:175:HIS:HB3	1.87	0.56
1:G:208:VAL:HG13	1:G:243:ILE:HD12	1.87	0.56
1:G:249:SER:O	1:G:249:SER:OG	2.21	0.56
1:B:227:GLN:O	1:B:227:GLN:HG3	2.06	0.56
1:G:130:THR:HG23	1:G:147:LEU:HB2	1.86	0.56
1:G:197:PRO:HA	1:G:224:LEU:HD13	1.88	0.56
1:G:285:ASN:O	1:G:289:THR:HG23	2.06	0.56

Continued on next page...

CONFIDENTIAL VALIDATION REPORT

Continued from previous page...

Atom-1	Atom-2	Interatomic distance (Å)	Clash overlap (Å)
1:H:212:CYS:HB2	1:H:219:PRO:HD3	1.88	0.56
1:C:165:GLU:HG3	1:C:268:ALA:HB3	1.88	0.56
1:D:164:GLU:OE1	1:D:269:LYS:HE3	2.06	0.56
1:B:251:VAL:HG12	1:B:253:VAL:HG23	1.86	0.56
1:F:183:ILE:HG22	1:F:184:SER:N	2.21	0.56
1:A:225:ALA:HB3	1:A:231:VAL:HG23	1.87	0.55
1:H:228:ILE:HD12	1:H:234:LEU:HD11	1.88	0.55
1:C:92:VAL:CG2	1:B:224:LEU:HD11	2.34	0.55
1:E:100:SER:HB3	1:E:228:ILE:HD12	1.87	0.55
1:E:233:ASN:HB3	1:F:102:ALA:HB1	1.88	0.55
1:H:158:HIS:O	1:H:273:ALA:HA	2.06	0.55
1:C:197:PRO:CB	1:C:224:LEU:HD11	2.36	0.55
1:D:280:SER:HB3	1:D:283:LEU:HB3	1.87	0.55
1:A:100:SER:HB3	1:A:228:ILE:HD12	1.87	0.55
1:E:232:ILE:HD13	1:E:258:TYR:OH	2.07	0.55
1:C:118:TYR:CE2	1:C:285:ASN:HB3	2.42	0.55
1:C:144:VAL:HG22	1:C:272:LEU:HD11	1.89	0.55
1:F:200:ILE:CG2	1:F:204:LEU:HB3	2.36	0.55
1:B:129:ASN:O	1:B:133:LEU:HG	2.06	0.54
1:G:183:ILE:HG21	1:G:188:PHE:HE1	1.71	0.54
1:B:185:LEU:HG	1:B:261:ILE:HG21	1.89	0.54
1:E:129:ASN:HB2	1:E:132:ARG:H	1.72	0.54
1:C:139:GLU:OE1	1:B:198:ARG:CG	2.47	0.54
1:C:231:VAL:O	1:C:235:VAL:HG23	2.08	0.54
1:H:180:TYR:O	1:H:259:ARG:NH1	2.30	0.54
1:B:249:SER:HA	1:B:258:TYR:CE2	2.43	0.54
1:E:97:PHE:CE1	1:E:127:GLU:HB2	2.42	0.54
1:E:107:VAL:HB	1:E:108:PRO:HD3	1.90	0.54
1:E:247:SER:O	1:E:250:GLN:HG3	2.08	0.54
1:A:158:HIS:CE1	1:A:276:ARG:HA	2.43	0.54
1:A:250:GLN:OE1	1:B:254:ILE:HG13	2.07	0.53
1:H:144:VAL:HG22	1:H:272:LEU:HD11	1.90	0.53
1:B:175:HIS:CD2	1:B:176:PRO:HD2	2.44	0.53
1:G:175:HIS:CD2	1:G:176:PRO:HD2	2.43	0.53
1:H:167:MET:HG3	1:H:245:PRO:HA	1.90	0.53
1:C:265:THR:O	1:C:265:THR:OG1	2.22	0.53
1:E:195:LEU:HD21	1:E:208:VAL:HG11	1.91	0.53
1:A:118:TYR:HB3	1:A:121:VAL:HG23	1.90	0.53
1:G:158:HIS:O	1:G:273:ALA:HA	2.09	0.53
1:D:92:VAL:HG23	1:D:122:ARG:O	2.08	0.53
1:B:198:ARG:HD2	1:B:198:ARG:N	2.22	0.53

Continued on next page...

CONFIDENTIAL VALIDATION REPORT

Continued from previous page...

Atom-1	Atom-2	Interatomic distance (Å)	Clash overlap (Å)
1:D:253:VAL:O	1:D:256:VAL:HG22	2.09	0.52
1:F:137:LEU:O	1:F:275:ARG:NH1	2.42	0.52
1:H:189:ARG:HB2	1:H:217:PHE:CG	2.44	0.52
1:D:132:ARG:HG2	1:D:132:ARG:HH21	1.74	0.52
1:A:107:VAL:HB	1:A:108:PRO:HD3	1.92	0.52
1:C:130:THR:HG23	1:C:147:LEU:HB2	1.91	0.52
1:A:149:PRO:HD3	1:A:271:ALA:HB3	1.91	0.52
1:D:128:ASP:OD1	1:D:129:ASN:N	2.42	0.52
1:C:112:ARG:HG3	1:D:238:GLU:HB2	1.90	0.52
1:A:189:ARG:HA	1:A:217:PHE:CE2	2.45	0.52
1:B:235:VAL:HG12	1:B:256:VAL:HG12	1.92	0.52
1:G:217:PHE:HE2	1:G:219:PRO:HB3	1.74	0.52
1:D:169:ILE:HG22	1:D:259:ARG:O	2.09	0.52
1:A:183:ILE:HG23	1:A:188:PHE:HE2	1.75	0.52
1:D:225:ALA:CB	1:B:225:ALA:HB3	2.39	0.52
1:B:198:ARG:N	1:B:198:ARG:CD	2.72	0.52
1:B:198:ARG:NE	1:B:227:GLN:HB3	2.24	0.52
1:C:157:PHE:HB3	1:C:274:TYR:O	2.09	0.51
1:C:160:ARG:HG3	1:C:160:ARG:HH11	1.74	0.51
1:A:127:GLU:OE1	1:B:228:ILE:HG23	2.10	0.51
1:A:149:PRO:HD3	1:A:271:ALA:CB	2.40	0.51
1:H:158:HIS:ND1	1:H:276:ARG:HA	2.24	0.51
1:H:250:GLN:O	1:H:252:LYS:N	2.43	0.51
1:D:104:ASN:OD1	1:D:106:VAL:HG13	2.10	0.51
1:A:88:GLY:HA3	1:A:281:PRO:HB2	1.93	0.51
1:E:112:ARG:HD3	1:F:238:GLU:CG	2.41	0.51
1:F:195:LEU:HD21	1:F:208:VAL:CG1	2.41	0.51
1:A:189:ARG:HA	1:A:217:PHE:CD2	2.45	0.51
1:F:132:ARG:HA	1:F:135:ASP:HB3	1.92	0.51
1:F:193:PHE:HB3	1:F:195:LEU:HD13	1.93	0.51
1:B:251:VAL:O	1:B:253:VAL:N	2.41	0.51
1:F:168:MET:HE3	1:F:260:HIS:NE2	2.26	0.51
1:C:125:LEU:HD11	1:D:239:MET:SD	2.50	0.51
1:D:228:ILE:HD11	1:D:234:LEU:HD11	1.93	0.51
1:D:244:VAL:HB	1:D:248:MET:HG3	1.92	0.50
1:B:177:ALA:HB1	1:B:183:ILE:HD11	1.93	0.50
1:H:225:ALA:HB3	1:H:226:PRO:CD	2.24	0.50
1:C:167:MET:HG3	1:C:266:PRO:O	2.12	0.50
1:D:208:VAL:HG13	1:D:243:ILE:HD12	1.94	0.50
1:A:259:ARG:HG3	1:A:259:ARG:HH11	1.76	0.50
1:C:113:ALA:O	1:C:116:ARG:HB3	2.12	0.50

Continued on next page...

CONFIDENTIAL VALIDATION REPORT

Continued from previous page...

Atom-1	Atom-2	Interatomic distance (Å)	Clash overlap (Å)
1:E:92:VAL:HG21	1:H:224:LEU:CD1	2.38	0.50
1:G:160:ARG:HG3	1:G:160:ARG:NH1	2.27	0.50
1:B:96:GLY:HA2	1:B:126:GLU:O	2.12	0.50
1:F:94:ARG:CG	1:F:124:GLN:HB3	2.41	0.50
1:H:213:ARG:HE	1:H:218:GLU:CD	2.14	0.49
1:D:147:LEU:HD13	1:D:151:PHE:CE2	2.46	0.49
1:C:151:PHE:CD1	1:C:151:PHE:C	2.86	0.49
1:E:205:TYR:O	1:E:209:ILE:HG12	2.11	0.49
1:F:107:VAL:HB	1:F:108:PRO:HD3	1.94	0.49
1:A:185:LEU:HD11	1:A:261:ILE:HD13	1.94	0.49
1:F:165:GLU:HG3	1:F:268:ALA:HB3	1.93	0.49
1:F:183:ILE:O	1:F:262:ALA:N	2.27	0.49
1:H:165:GLU:OE1	1:H:247:SER:OG	2.23	0.49
1:F:232:ILE:HD13	1:F:258:TYR:OH	2.12	0.49
1:C:236:ALA:O	1:D:112:ARG:NH2	2.46	0.49
1:G:170:VAL:HG13	1:G:235:VAL:HG21	1.94	0.49
1:F:94:ARG:HD2	1:F:141:SER:O	2.12	0.49
1:F:211:SER:OG	1:F:266:PRO:HD3	2.12	0.49
1:C:103:PHE:HA	1:D:233:ASN:HD21	1.77	0.49
1:C:115:ARG:HD2	1:C:123:LEU:HD12	1.93	0.49
1:H:247:SER:O	1:H:250:GLN:OE1	2.31	0.49
1:C:89:GLU:HG2	1:C:118:TYR:CE1	2.47	0.49
1:C:209:ILE:HD13	1:C:209:ILE:N	2.27	0.49
1:D:166:PRO:HA	1:D:267:THR:HG22	1.94	0.49
1:B:232:ILE:HD13	1:B:258:TYR:OH	2.13	0.49
1:H:189:ARG:HA	1:H:217:PHE:CE2	2.48	0.49
1:G:209:ILE:O	1:G:213:ARG:HG3	2.13	0.48
1:E:254:ILE:HG13	1:F:250:GLN:OE1	2.13	0.48
1:F:265:THR:HB	1:F:266:PRO:CD	2.43	0.48
1:F:251:VAL:HG12	1:F:253:VAL:HG23	1.95	0.48
1:B:189:ARG:HA	1:B:217:PHE:CD2	2.49	0.48
1:G:127:GLU:CD	1:H:228:ILE:HG23	2.34	0.48
1:G:170:VAL:HG22	1:G:235:VAL:HG11	1.95	0.48
1:C:252:LYS:O	1:D:250:GLN:HB3	2.13	0.48
1:A:231:VAL:O	1:A:235:VAL:HG23	2.14	0.48
1:H:276:ARG:HG2	1:H:276:ARG:NH1	2.28	0.47
1:C:183:ILE:HD13	1:C:183:ILE:HA	1.71	0.47
1:E:105:SER:O	1:E:108:PRO:HD2	2.14	0.47
1:G:175:HIS:CG	1:G:176:PRO:HD2	2.49	0.47
1:H:202:LEU:HA	1:H:202:LEU:HD23	1.47	0.47
1:C:188:PHE:CE2	1:C:241:VAL:HG11	2.49	0.47

Continued on next page...

CONFIDENTIAL VALIDATION REPORT

Continued from previous page...

Atom-1	Atom-2	Interatomic distance (Å)	Clash overlap (Å)
1:F:141:SER:O	1:F:142:LEU:HD23	2.14	0.47
1:C:96:GLY:HA2	1:C:126:GLU:O	2.14	0.47
1:D:137:LEU:O	1:D:275:ARG:NH1	2.47	0.47
1:C:234:LEU:O	1:C:239:MET:HB2	2.14	0.47
1:F:265:THR:HB	1:F:266:PRO:HD2	1.96	0.47
1:G:250:GLN:OE1	1:H:253:VAL:HA	2.14	0.47
1:A:94:ARG:HB3	1:A:142:LEU:HA	1.96	0.47
1:B:283:LEU:O	1:B:287:VAL:HG23	2.15	0.47
1:G:201:GLY:O	1:G:205:TYR:HB3	2.15	0.47
1:C:251:VAL:HG12	1:C:253:VAL:HG23	1.97	0.47
1:D:202:LEU:CD2	1:A:140:GLY:HA3	2.31	0.47
1:E:96:GLY:HA2	1:E:126:GLU:O	2.14	0.47
1:G:147:LEU:HD13	1:G:151:PHE:HE2	1.79	0.46
1:H:183:ILE:HD12	1:H:259:ARG:HB2	1.96	0.46
1:H:200:ILE:CG2	1:H:204:LEU:HB3	2.45	0.46
1:H:282:VAL:O	1:H:282:VAL:HG12	2.14	0.46
1:A:182:GLU:OE2	1:A:262:ALA:HB3	2.15	0.46
1:H:111:ILE:HG23	1:H:123:LEU:CD1	2.45	0.46
1:C:211:SER:HB3	1:C:266:PRO:HG2	1.97	0.46
1:E:97:PHE:O	1:E:127:GLU:HA	2.15	0.46
1:C:265:THR:N	1:C:266:PRO:CD	2.78	0.46
1:A:165:GLU:O	1:A:267:THR:HB	2.16	0.46
1:F:188:PHE:O	1:F:191[B]:GLU:HB2	2.15	0.46
1:H:199:GLU:HG3	1:H:229:ALA:CB	2.44	0.46
1:C:185:LEU:HB2	1:C:263:ASP:OD2	2.14	0.46
1:D:158:HIS:CE1	1:D:276:ARG:NH2	2.84	0.46
1:D:258:TYR:C	1:D:259:ARG:HD2	2.35	0.46
1:A:272:LEU:HD23	1:A:291:PHE:CE1	2.40	0.46
1:G:198:ARG:HD3	1:G:205:TYR:CD1	2.51	0.46
1:C:177:ALA:O	1:C:183:ILE:HD11	2.16	0.46
1:F:115:ARG:HG2	1:F:123:LEU:HD12	1.98	0.46
1:G:267:THR:OG1	1:G:269:LYS:NZ	2.49	0.46
1:F:200:ILE:HG23	1:F:204:LEU:HD23	1.97	0.46
1:F:202:LEU:O	1:F:202:LEU:HD23	2.16	0.45
1:E:144:VAL:HG23	1:E:274:TYR:HB3	1.97	0.45
1:F:231:VAL:O	1:F:235:VAL:HG23	2.17	0.45
1:H:283:LEU:HD12	1:H:283:LEU:O	2.16	0.45
1:E:112:ARG:CD	1:F:238:GLU:HG3	2.45	0.45
1:C:185:LEU:HG	1:C:261:ILE:CG2	2.46	0.45
1:B:196:PHE:HB3	1:B:198:ARG:NH2	2.32	0.45
1:H:195:LEU:HB3	1:H:205:TYR:HE1	1.80	0.45

Continued on next page...

CONFIDENTIAL VALIDATION REPORT

Continued from previous page...

Atom-1	Atom-2	Interatomic distance (Å)	Clash overlap (Å)
1:B:120:ASP:OD2	1:H:282:VAL:CG2	2.62	0.45
1:A:118:TYR:CZ	1:A:285:ASN:HB3	2.52	0.45
1:C:251:VAL:O	1:C:258:TYR:OH	2.27	0.45
1:A:95:VAL:HG22	1:A:144:VAL:HG12	1.99	0.45
1:F:132:ARG:HG2	1:F:132:ARG:NH2	2.31	0.45
1:D:132:ARG:HG2	1:D:132:ARG:NH2	2.32	0.45
1:E:171:MET:CE	1:E:259:ARG:HG2	2.47	0.44
1:G:96:GLY:HA2	1:G:126:GLU:O	2.17	0.44
1:H:195:LEU:HB3	1:H:205:TYR:CE1	2.52	0.44
1:F:200:ILE:HD11	1:F:244:VAL:HG12	1.99	0.44
1:G:112:ARG:HG3	1:H:238:GLU:OE1	2.17	0.44
1:E:89:GLU:O	1:E:90:LEU:HD23	2.18	0.44
1:F:205:TYR:OH	1:G:94:ARG:NH2	2.48	0.44
1:C:114:PHE:CD1	1:C:286:PHE:HA	2.52	0.44
1:E:125:LEU:HD11	1:F:239:MET:SD	2.58	0.44
1:F:189:ARG:HA	1:F:217:PHE:CD2	2.52	0.44
1:E:219:PRO:HG2	1:E:221:ILE:HD11	1.99	0.44
1:G:205:TYR:CE1	1:G:209:ILE:HD12	2.53	0.44
1:A:162:LEU:HD12	1:A:162:LEU:HA	1.79	0.44
1:B:107:VAL:HB	1:B:108:PRO:HD3	1.98	0.44
1:F:195:LEU:HD21	1:F:208:VAL:HG12	2.00	0.44
1:F:244:VAL:HB	1:F:248:MET:HG3	2.00	0.44
1:E:162:LEU:HA	1:E:162:LEU:HD12	1.79	0.44
1:F:263:ASP:O	1:F:265:THR:HG23	2.17	0.44
1:D:158:HIS:O	1:D:273:ALA:HA	2.18	0.44
1:G:275:ARG:NH2	1:G:278:ASP:OD2	2.50	0.44
1:D:148:ARG:NH1	1:D:269:LYS:O	2.51	0.44
1:A:137:LEU:HD23	1:A:137:LEU:HA	1.83	0.44
1:E:204:LEU:O	1:E:208:VAL:HG23	2.18	0.44
1:G:228:ILE:HA	1:G:231:VAL:HG23	2.00	0.43
1:A:110:ALA:HB1	1:A:290:VAL:HG22	2.00	0.43
1:F:169:ILE:HD13	1:F:188:PHE:CZ	2.52	0.43
1:G:159:LEU:HD12	1:G:273:ALA:HB2	1.99	0.43
1:D:200:ILE:HG22	1:D:204:LEU:HB3	1.99	0.43
1:F:182:GLU:HG2	1:F:262:ALA:HB2	1.99	0.43
1:F:237:ALA:O	1:F:238:GLU:HB2	2.18	0.43
1:H:232:ILE:O	1:H:235:VAL:HB	2.17	0.43
1:A:103:PHE:HB2	1:A:248:MET:HE2	2.01	0.43
1:G:159:LEU:HD12	1:G:159:LEU:HA	1.84	0.43
1:H:110:ALA:HB1	1:H:290:VAL:HG22	1.99	0.43
1:C:127:GLU:HB3	1:D:228:ILE:CG2	2.48	0.43

Continued on next page...

CONFIDENTIAL VALIDATION REPORT

Continued from previous page...

Atom-1	Atom-2	Interatomic distance (Å)	Clash overlap (Å)
1:H:228:ILE:HG22	1:H:230:SER:H	1.83	0.43
1:E:159:LEU:HA	1:E:159:LEU:HD12	1.60	0.43
1:F:144:VAL:HB	1:F:283:LEU:HD13	2.00	0.43
1:C:194:LEU:HD23	1:C:194:LEU:HA	1.80	0.43
1:H:169:ILE:HD13	1:H:169:ILE:HG21	1.82	0.43
1:B:93:LEU:HB3	1:B:123:LEU:HD23	2.00	0.43
1:E:251:VAL:HG12	1:E:253:VAL:HG23	2.00	0.43
1:C:235:VAL:HG13	1:C:240:GLY:O	2.19	0.43
1:A:161:MET:HG3	1:A:162:LEU:N	2.33	0.43
1:A:251:VAL:HG12	1:A:253:VAL:HG23	2.01	0.43
1:G:171:MET:HE2	1:G:178:ALA:N	2.34	0.43
1:C:92:VAL:HG23	1:C:122:ARG:O	2.18	0.42
1:D:194:LEU:HD23	1:D:194:LEU:HA	1.66	0.42
1:B:198:ARG:CD	1:B:198:ARG:H	2.32	0.42
1:H:168:MET:HE3	1:H:249:SER:HB3	2.01	0.42
1:F:104:ASN:OD1	1:F:106:VAL:HG13	2.18	0.42
1:C:90:LEU:HD23	1:C:91:GLY:N	2.34	0.42
1:B:194:LEU:HD13	1:B:234:LEU:HB2	2.00	0.42
1:E:109:THR:HG22	1:E:112:ARG:HH21	1.84	0.42
1:C:127:GLU:O	1:C:127:GLU:HG2	2.15	0.42
1:F:181:GLU:CD	1:F:181:GLU:H	2.23	0.42
1:A:233:ASN:HB3	1:B:102:ALA:HB1	2.01	0.42
1:F:89:GLU:O	1:F:120:ASP:HB2	2.20	0.42
1:H:288:LEU:HD23	1:H:288:LEU:HA	1.87	0.42
1:E:227:GLN:O	1:E:230:SER:N	2.47	0.42
1:F:261:ILE:HG21	1:F:265:THR:CG2	2.50	0.42
1:D:185:LEU:HD23	1:D:185:LEU:HA	1.69	0.42
1:D:200:ILE:HG23	1:D:204:LEU:HD23	2.02	0.42
1:F:204:LEU:O	1:F:208:VAL:HG23	2.19	0.42
1:A:98:THR:N	1:A:101:SER:OG	2.53	0.42
1:G:148:ARG:HG2	1:G:148:ARG:HH11	1.84	0.42
1:H:267:THR:OG1	1:H:269:LYS:HE3	2.19	0.42
1:B:185:LEU:HA	1:B:185:LEU:HD23	1.88	0.42
1:H:213:ARG:NH2	1:H:218:GLU:OE2	2.47	0.42
1:B:170:VAL:HG22	1:B:235:VAL:HG11	2.01	0.42
1:F:94:ARG:HB3	1:F:142:LEU:HA	2.02	0.42
1:H:259:ARG:HG3	1:H:259:ARG:HH11	1.85	0.42
1:D:167:MET:HG2	1:D:245:PRO:HA	2.02	0.41
1:G:252:LYS:O	1:H:250:GLN:HG2	2.19	0.41
1:A:103:PHE:HB2	1:A:248:MET:CE	2.51	0.41
1:A:151:PHE:H	1:A:151:PHE:HD1	1.67	0.41

Continued on next page...

CONFIDENTIAL VALIDATION REPORT

Continued from previous page...

Atom-1	Atom-2	Interatomic distance (Å)	Clash overlap (Å)
1:F:95:VAL:HG21	1:F:123:LEU:HD22	2.01	0.41
1:F:95:VAL:O	1:F:125:LEU:HA	2.20	0.41
1:C:221:ILE:HD13	1:C:221:ILE:HA	1.74	0.41
1:B:108:PRO:O	1:B:112:ARG:HB2	2.20	0.41
1:F:202:LEU:HD23	1:F:202:LEU:C	2.40	0.41
1:F:272:LEU:HD23	1:F:291:PHE:HE2	1.85	0.41
1:A:234:LEU:HD23	1:A:234:LEU:HA	1.86	0.41
1:B:125:LEU:HA	1:B:125:LEU:HD23	1.68	0.41
1:B:258:TYR:O	1:B:259:ARG:HD3	2.19	0.41
1:G:225:ALA:HB3	1:G:231:VAL:HG22	2.02	0.41
1:H:158:HIS:CE1	1:H:276:ARG:HG2	2.54	0.41
1:D:95:VAL:HG13	1:D:144:VAL:HG23	2.01	0.41
1:A:183:ILE:HG23	1:A:188:PHE:CE2	2.55	0.41
1:B:204:LEU:HD21	1:B:245:PRO:HG3	2.02	0.41
1:G:236:ALA:HB2	1:G:256:VAL:HG13	2.01	0.41
1:B:101:SER:O	1:B:104:ASN:HB3	2.20	0.41
1:B:209:ILE:HG23	1:B:219:PRO:HG3	2.02	0.41
1:F:248:MET:C	1:F:250:GLN:H	2.24	0.41
1:F:270:LEU:HD12	1:F:270:LEU:HA	1.69	0.41
1:H:212:CYS:HB3	1:H:217:PHE:CE1	2.56	0.41
1:F:183:ILE:HD12	1:F:183:ILE:N	2.36	0.41
1:C:103:PHE:HA	1:D:233:ASN:ND2	2.35	0.41
1:E:185:LEU:HD23	1:E:185:LEU:HA	1.82	0.41
1:C:116:ARG:O	1:C:119:PRO:HD3	2.21	0.41
1:C:284:ARG:HA	1:C:287:VAL:HB	2.03	0.41
1:D:108:PRO:O	1:D:112:ARG:HB3	2.21	0.41
1:H:142:LEU:HA	1:H:142:LEU:HD23	1.79	0.41
1:B:128:ASP:OD1	1:B:129:ASN:N	2.48	0.41
1:B:165:GLU:OE1	1:B:247:SER:OG	2.30	0.40
1:E:258:TYR:O	1:E:259:ARG:HD3	2.21	0.40
1:H:282:VAL:O	1:H:282:VAL:CG1	2.68	0.40
1:C:148:ARG:NE	1:C:204:LEU:HD11	2.36	0.40
1:C:188:PHE:CD2	1:C:191:GLU:HG3	2.56	0.40
1:D:177:ALA:HB1	1:D:183:ILE:HD13	2.01	0.40
1:E:175:HIS:CG	1:E:176:PRO:HD2	2.56	0.40
1:G:86:VAL:N	1:G:89:GLU:OE2	2.54	0.40
1:C:124:GLN:HG3	1:B:224:LEU:CD1	2.51	0.40
1:D:118:TYR:CZ	1:D:285:ASN:ND2	2.89	0.40
1:D:205:TYR:OH	1:A:94:ARG:NH2	2.54	0.40
1:E:94:ARG:HG3	1:E:143:ASP:OD2	2.21	0.40
1:G:166:PRO:O	1:G:246:ALA:HB2	2.21	0.40

Continued on next page...

CONFIDENTIAL VALIDATION REPORT

Continued from previous page...

Atom-1	Atom-2	Interatomic distance (Å)	Clash overlap (Å)
1:C:151:PHE:CZ	1:C:154:SER:HA	2.56	0.40
1:A:98:THR:HG1	1:A:101:SER:CB	2.35	0.40
1:B:103:PHE:CD2	1:B:248:MET:HG2	2.56	0.40
1:E:104:ASN:OD1	1:E:106:VAL:HG22	2.21	0.40
1:F:236:ALA:HB2	1:F:256:VAL:HG13	2.04	0.40
1:G:188:PHE:HD2	1:G:191:GLU:HG2	1.85	0.40

There are no symmetry-related clashes.

5.3 Torsion angles (i)

5.3.1 Protein backbone (i)

In the following table, the Percentiles column shows the percent Ramachandran outliers of the chain as a percentile score with respect to all X-ray entries followed by that with respect to entries of similar resolution.

The Analysed column shows the number of residues for which the backbone conformation was analysed, and the total number of residues.

Mol	Chain	Analysed	Favoured	Allowed	Outliers	Percentiles	
1	A	198/292 (68%)	191 (96%)	7 (4%)	0	100	100
1	B	200/292 (68%)	190 (95%)	9 (4%)	1 (0%)	29	62
1	C	205/292 (70%)	192 (94%)	13 (6%)	0	100	100
1	D	202/292 (69%)	188 (93%)	13 (6%)	1 (0%)	29	62
1	E	205/292 (70%)	195 (95%)	10 (5%)	0	100	100
1	F	204/292 (70%)	193 (95%)	11 (5%)	0	100	100
1	G	205/292 (70%)	190 (93%)	15 (7%)	0	100	100
1	H	205/292 (70%)	193 (94%)	12 (6%)	0	100	100
All	All	1624/2336 (70%)	1532 (94%)	90 (6%)	2 (0%)	51	82

All (2) Ramachandran outliers are listed below:

Mol	Chain	Res	Type
1	D	226	PRO
1	B	226	PRO

CONFIDENTIAL VALIDATION REPORT

5.3.2 Protein sidechains ⓘ

In the following table, the Percentiles column shows the percent sidechain outliers of the chain as a percentile score with respect to all X-ray entries followed by that with respect to entries of similar resolution.

The Analysed column shows the number of residues for which the sidechain conformation was analysed, and the total number of residues.

Mol	Chain	Analysed	Rotameric	Outliers	Percentiles	
1	A	143/238 (60%)	142 (99%)	1 (1%)	84	90
1	B	158/238 (66%)	156 (99%)	2 (1%)	69	82
1	C	156/238 (66%)	152 (97%)	4 (3%)	46	71
1	D	158/238 (66%)	156 (99%)	2 (1%)	69	82
1	E	152/238 (64%)	151 (99%)	1 (1%)	84	90
1	F	166/238 (70%)	165 (99%)	1 (1%)	86	91
1	G	159/238 (67%)	155 (98%)	4 (2%)	47	71
1	H	161/238 (68%)	158 (98%)	3 (2%)	57	76
All	All	1253/1904 (66%)	1235 (99%)	18 (1%)	67	81

All (18) residues with a non-rotameric sidechain are listed below:

Mol	Chain	Res	Type
1	C	151	PHE
1	C	156	ARG
1	C	161	MET
1	C	180	TYR
1	D	199	GLU
1	D	274	TYR
1	A	97	PHE
1	B	263	ASP
1	B	284	ARG
1	E	129	ASN
1	F	284	ARG
1	G	127	GLU
1	G	156	ARG
1	G	174	ASN
1	G	249	SER
1	H	147	LEU
1	H	154	SER
1	H	269	LYS

CONFIDENTIAL VALIDATION REPORT

Sometimes sidechains can be flipped to improve hydrogen bonding and reduce clashes. All (12) such sidechains are listed below:

Mol	Chain	Res	Type
1	D	158	HIS
1	D	223	GLN
1	D	233	ASN
1	D	285	ASN
1	B	175	HIS
1	E	124	GLN
1	E	260	HIS
1	E	285	ASN
1	F	233	ASN
1	H	129	ASN
1	H	227	GLN
1	H	233	ASN

5.3.3 RNA (i)

There are no RNA molecules in this entry.

5.4 Non-standard residues in protein, DNA, RNA chains (i)

There are no non-standard protein/DNA/RNA residues in this entry.

5.5 Carbohydrates (i)

There are no monosaccharides in this entry.

5.6 Ligand geometry (i)

There are no ligands in this entry.

5.7 Other polymers (i)

There are no such residues in this entry.

5.8 Polymer linkage issues (i)

There are no chain breaks in this entry.

CONFIDENTIAL VALIDATION REPORT

6 Fit of model and data (i)

6.1 Protein, DNA and RNA chains (i)

In the following table, the column labelled '#RSRZ> 2' contains the number (and percentage) of RSRZ outliers, followed by percent RSRZ outliers for the chain as percentile scores relative to all X-ray entries and entries of similar resolution. The OWAB column contains the minimum, median, 95th percentile and maximum values of the occupancy-weighted average B-factor per residue. The column labelled 'Q< 0.9' lists the number of (and percentage) of residues with an average occupancy less than 0.9.

Mol	Chain	Analysed	<RSRZ>	#RSRZ>2			OWAB(Å^2)	Q<0.9
1	A	204/292 (69%)	-0.18	2 (0%)	82	82	18, 44, 83, 96	0
1	B	202/292 (69%)	-0.31	0	100	100	19, 38, 60, 83	0
1	C	207/292 (70%)	-0.23	4 (1%)	66	64	21, 44, 76, 100	0
1	D	204/292 (69%)	-0.33	0	100	100	19, 39, 61, 76	0
1	E	207/292 (70%)	-0.11	3 (1%)	75	74	27, 45, 81, 93	0
1	F	205/292 (70%)	-0.24	2 (0%)	82	82	21, 40, 69, 83	0
1	G	207/292 (70%)	-0.21	2 (0%)	82	82	25, 45, 82, 93	0
1	H	207/292 (70%)	-0.19	3 (1%)	75	74	22, 42, 67, 98	0
All	All	1643/2336 (70%)	-0.22	16 (0%)	82	82	18, 42, 77, 100	0

All (16) RSRZ outliers are listed below:

Mol	Chain	Res	Type	RSRZ
1	E	292	PRO	4.7
1	H	182	GLU	4.4
1	H	264	GLN	3.8
1	C	86	VAL	3.6
1	C	179	SER	3.4
1	A	201	GLY	3.4
1	F	180	TYR	3.3
1	H	179	SER	2.9
1	C	181	GLU	2.9
1	G	263	ASP	2.8
1	E	86	VAL	2.7
1	C	265	THR	2.5
1	A	198	ARG	2.4
1	E	197	PRO	2.4
1	F	264	GLN	2.3
1	G	264	GLN	2.2

CONFIDENTIAL VALIDATION REPORT

6.2 Non-standard residues in protein, DNA, RNA chains (i)

There are no non-standard protein/DNA/RNA residues in this entry.

6.3 Carbohydrates (i)

There are no monosaccharides in this entry.

6.4 Ligands (i)

There are no ligands in this entry.

6.5 Other polymers (i)

There are no such residues in this entry.

CONFIDENTIAL VALIDATION REPORT
