# Supplementary material for: A genetically encoded fluorescent biosensor for detecting itaconate with subcellular resolution in living macrophages
Source: Nat Commun. 2022 Nov 4;13:6562. doi: 10.1038/s41467-022-34306-5 (PMC9636186; doi:10.1038/s41467-022-34306-5)

Supplementary Fig. 3b

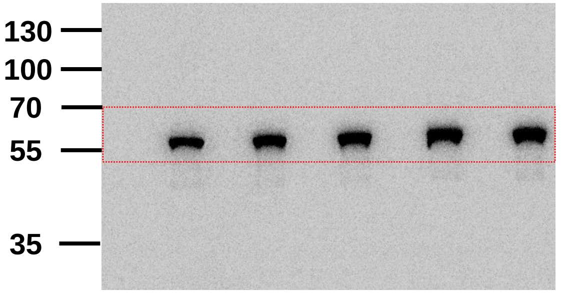

WB: IRG1

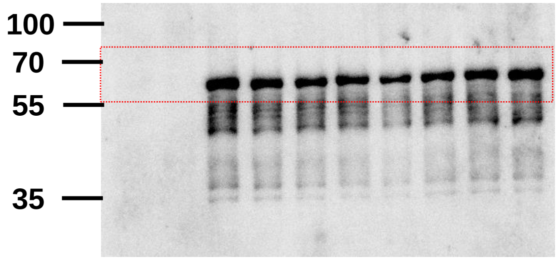

WB: GFP (BioITA)

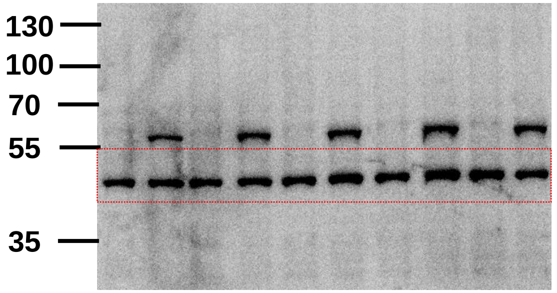

WB: β-actin

Supplementary Fig. 4b

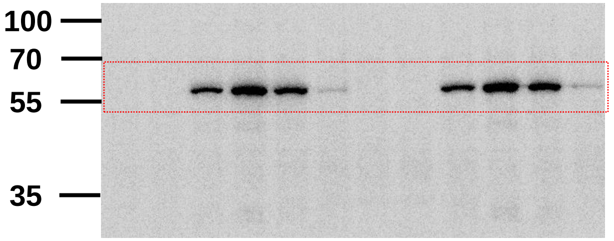

WB: IRG1

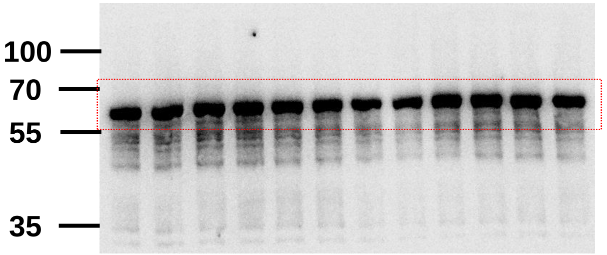

WB: GFP (BioITA)

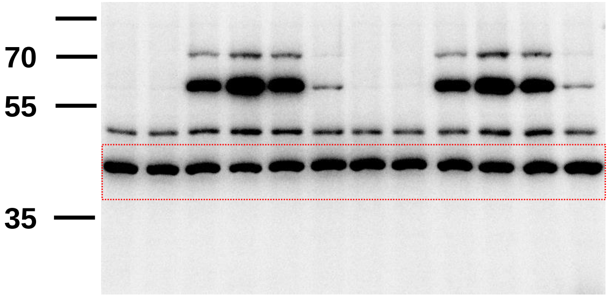

WB: β-actin

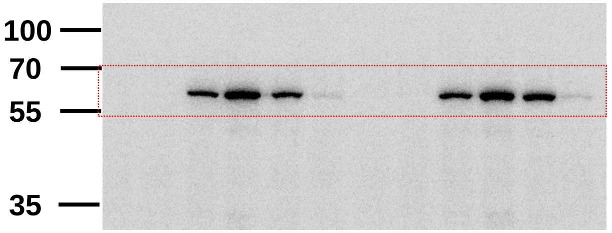

WB: IRG1

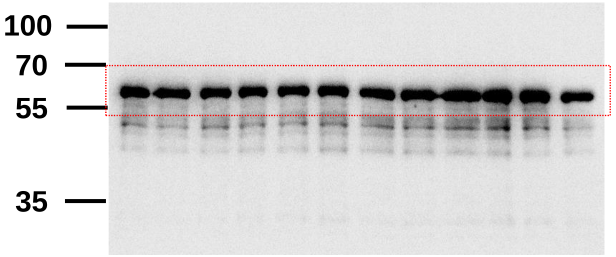

WB: GFP (BioITA)

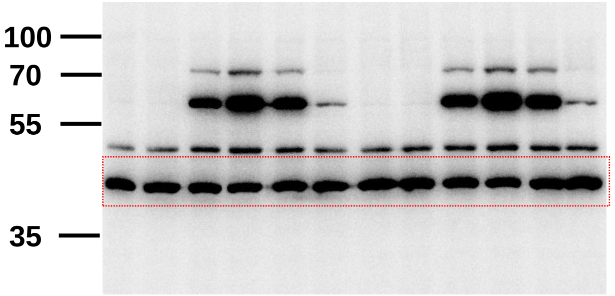

WB: β-actin

Supplementary Fig. 4f

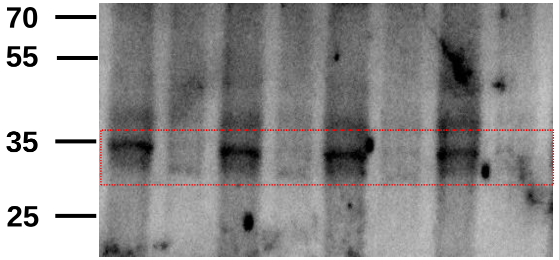

WB: OGC

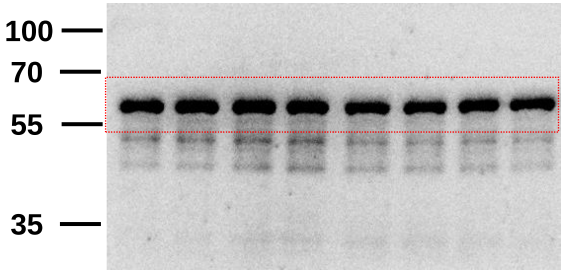

WB: GFP (BioITA)

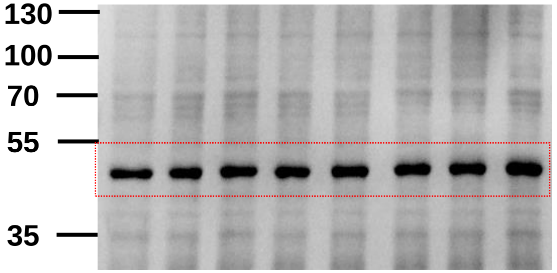

WB: β-actin

Supplementary Fig. 5a

Supplementary Fig. 5c

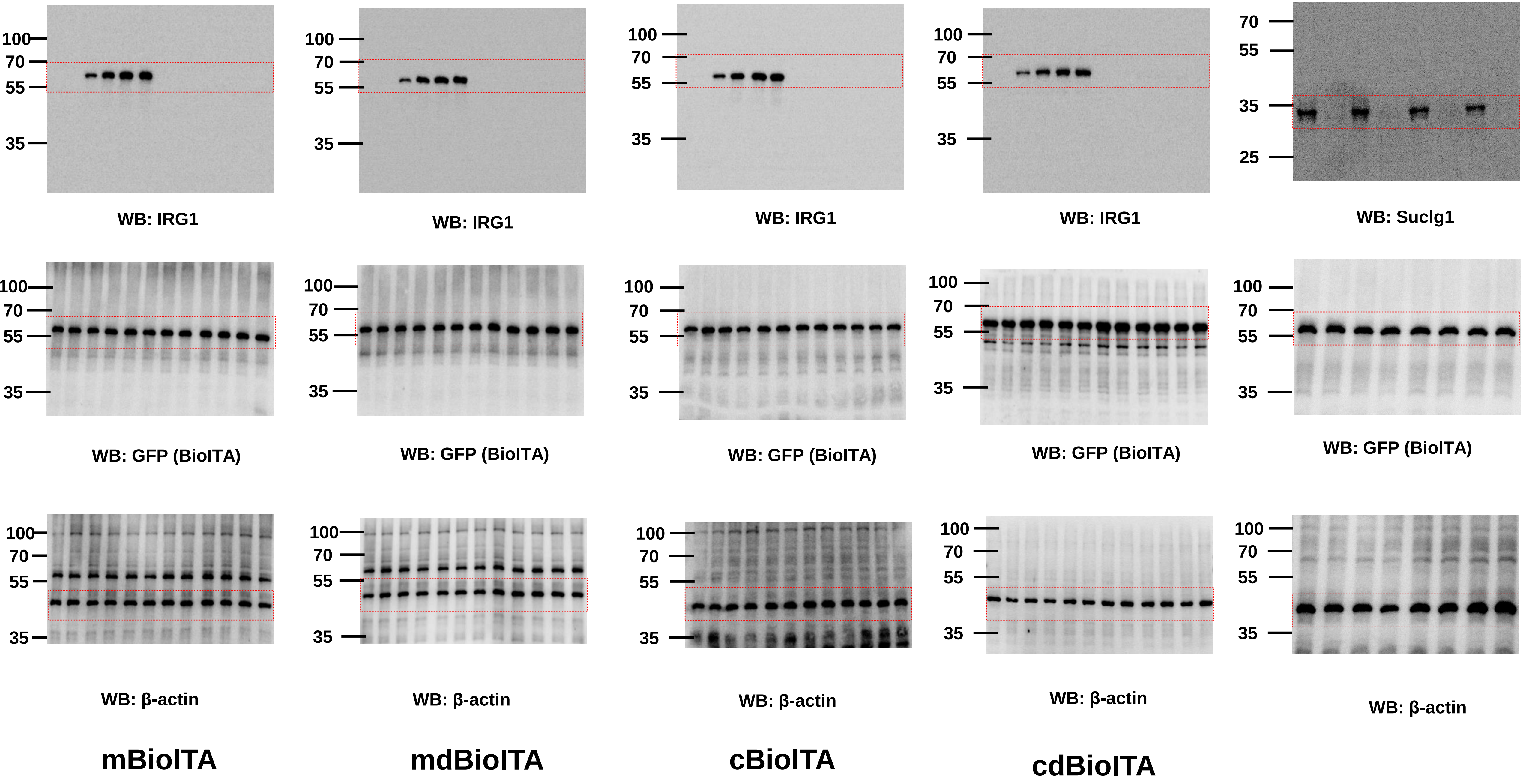

Supplementary Fig. 6b

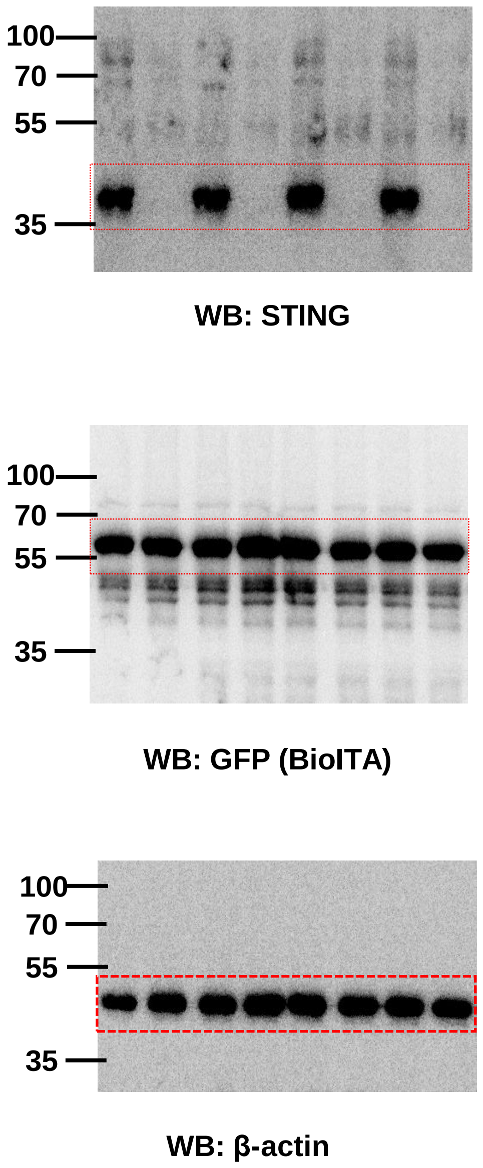

Supplementary Fig. 6c

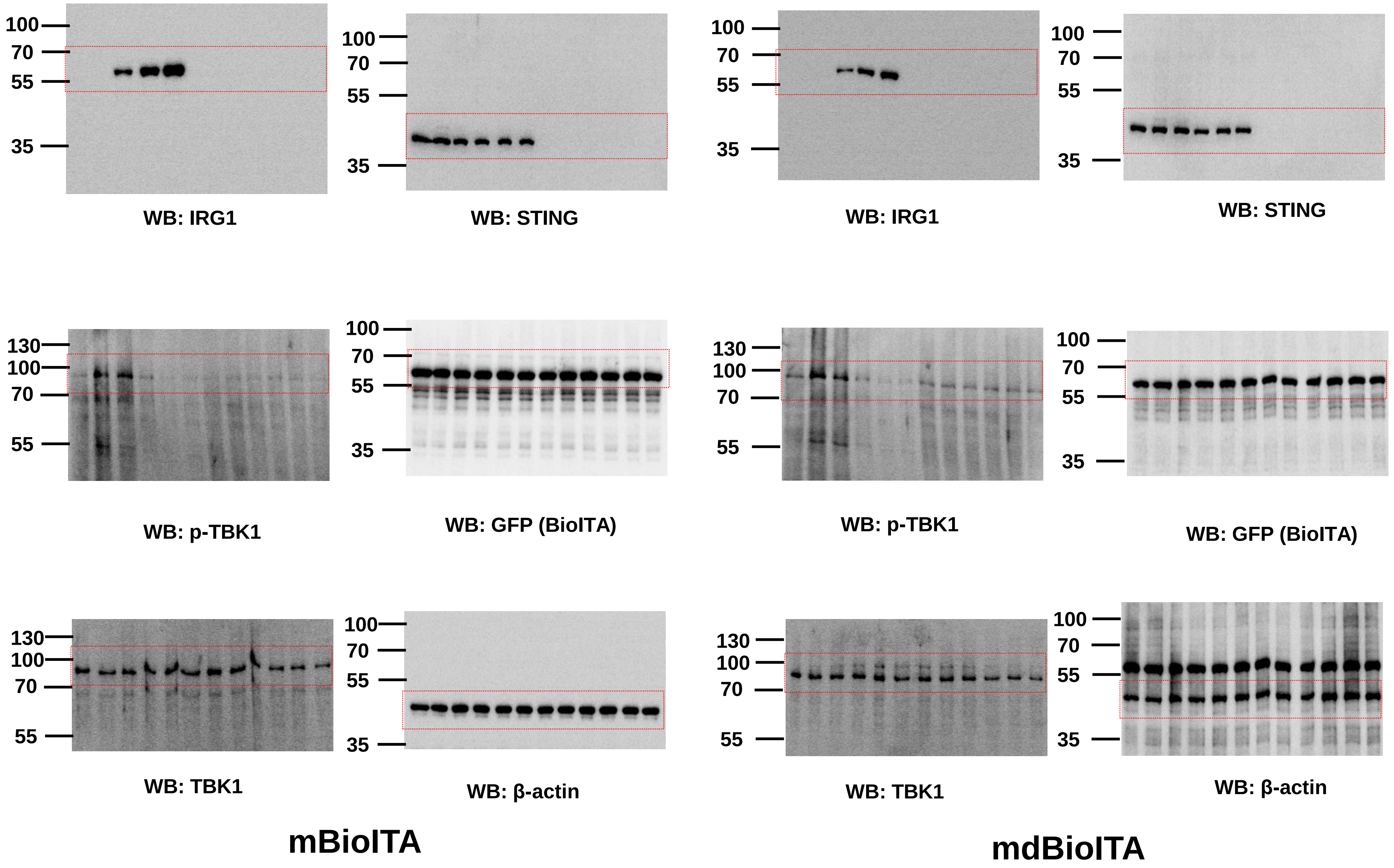

Supplementary Fig. 6c

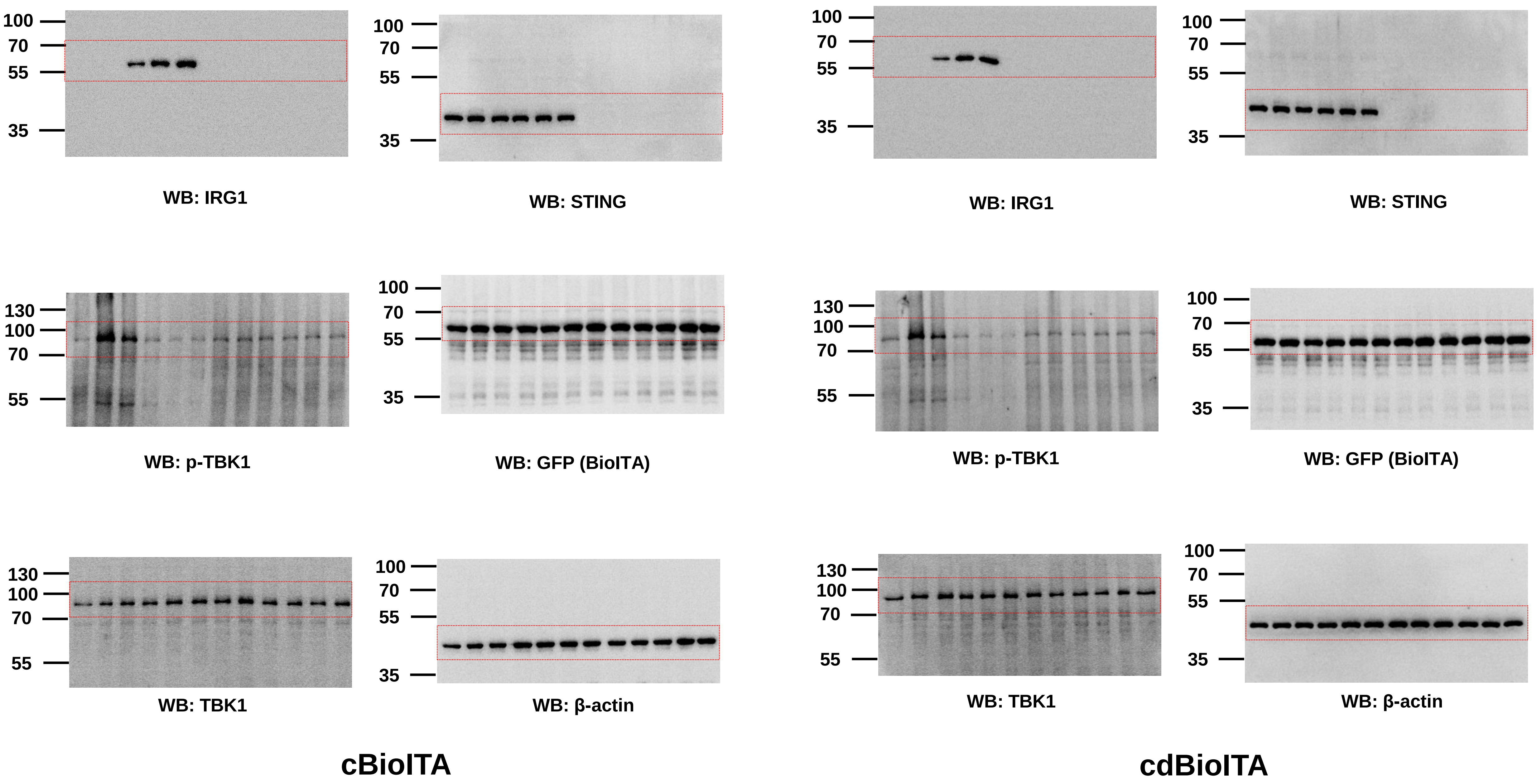

Supplementary Fig. 7c

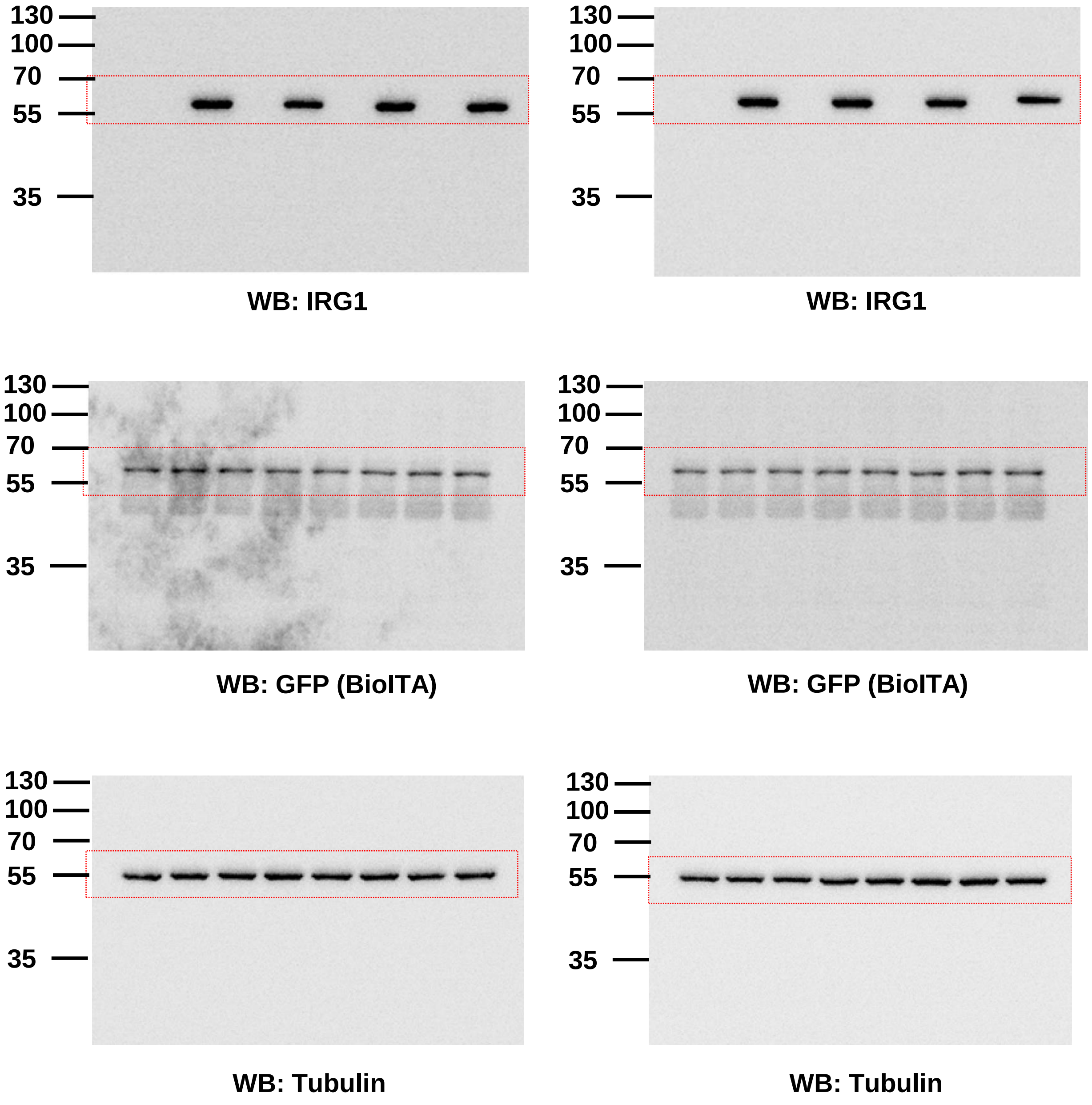

Supplement: Supplementary file 4 — Source Data [file 41467_2022_34306_MOESM4_ESM.zip › Unprocessed gels/Unprocessed gels BioITA.pdf]
